# Comparative Analysis of Clinical and Environmental Strains of *Exophiala spinifera* by Long-Reads Sequencing and RNAseq Reveal Adaptive Strategies

**DOI:** 10.3389/fmicb.2020.01880

**Published:** 2020-07-31

**Authors:** Yinggai Song, Minghao Du, Nickolas Menezes da Silva, Ence Yang, Vania A. Vicente, G. Sybren de Hoog, Ruoyu Li

**Affiliations:** ^1^Department of Dermatology and Venerology, Peking University First Hospital, Beijing, China; ^2^Research Center for Medical Mycology, Peking University, Beijing, China; ^3^National Clinical Research Center for Skin and Immune Diseases, Beijing, China; ^4^Peking University Health Science Center, Beijing, China; ^5^Microbiology, Parasitology and Pathology Post-Graduation Program, Department of Pathology, Federal University of Paraná, Curitiba, Brazil; ^6^Center of Expertise in Mycology of Radboud University Medical Center, Canisius Wilhelmina Hospital, Nijmegen, Netherlands

**Keywords:** black yeasts, comparative genomics, transcriptome analysis, intraspecific variability, gene rearrangement, CARD9 deficiency, pathogenicity, virulence

## Abstract

*Exophiala spinifera*, a capsule-producing black yeast, is overrepresented as agent of disseminated infection in humans with inherited dysfunction of the *CARD9* gene. In a review of published caspase recruitment domain-containing protein 9 (CARD9) deficiency cases, black fungi were linked to mutations other than those prevalent in yeast and dermatophyte cases, and were found to respond to a larger panel of cytokines. Here, we sequenced and annotated the genomes of BMU 08022 from a patient with CARD9 deficiency and two environmental strains, BMU 00051 and BMU 00047. We performed genomic and transcriptomic analysis for these isolates including published black yeasts genomes, using a combination of long-read (PACBIO) and short-read (Illumina) sequencing technologies with a hybrid assembly strategy. We identified the virulence factors, fitness, and the major genetic and gene expression differences between the strains with RNAseq technology. Genome assembly reached sub-chromosome level with between 12,043 and 12,130 predicted genes. The number of indels identified in the clinical strain was higher than observed in environmental strains. We identify a relatively large core genome of 9,887 genes. Moreover, substantial syntenic rearrangements of scaffolds I and III in the *CARD9*-related isolate were detected. Seventeen gene clusters were involved in the production of secondary metabolites. PKS-cluster 17 was consistently found to be absent in the clinical strain. Comparative transcriptome analysis demonstrated that 16 single-copy genes were significantly differentially expressed upon incubation in brain-heart infusion broth vs. Sabouraud glucose broth. Most of the single-copy genes upregulated with Brain Heart Infusion (BHI) were transporters. There were 48 unique genes differentially expressed exclusively to the clinical strain in two different media, including genes from various metabolic processes and transcriptional regulation. Up-regulated genes in the clinical strain with Gene Ontology (GO) enrichment are mainly involved in transmembrane transport, biosynthetic process and metabolic process. This study has provided novel insights into understanding of strain-differences in intrinsic virulence of the species and indicated that intraspecific variability may be related to habitat choice. This indicates that strains of *E. spinifera* are differentially prone to cause infection in susceptible patient populations, and provides clues for future studies exploring the mechanisms of pathogenic and adaptive strategies of black yeasts in immunodeficient patients.

## Introduction

Melanized fungi of the order Chaetothyriales are renowned as agents of human infection. The number of cases is low when compared to dermatophytes, *Aspergillus*, and *Candida*, but with 86 species in 7 genera that have been proven as potential agents of vertebrate disease (de Hoog et al., 2020) the order ranges fourth in clinical biodiversity, after Onygenales, Eurotiales, and Saccharomycetales. In 19 chaetothyrialean species, systemic or disseminated infection has been observed, which frequently led to death of the patient. The neurotropic species *Cladophialophora bantiana* is one of the most feared fungi, because it causes brain abscesses in healthy-appearing patients, with a case fatality rate of 65% despite antifungal therapy (Kantarcioglu et al., 2017). Numerous investigations nevertheless suggest that most members of the order – with a possible exception of agents of chromoblastomycosis – are opportunists rather than pathogens. Opportunists are defined as fungi that complete their natural life cycle without involvement of an animal host, being able of unintentional infection only through coincidental similarities between conditions of host tissue and natural habitat. Gostincar et al. (2018) ascribed their infectious ability to polyextremotolerance, which is tolerance to environmental stress supplemented with efficient nutrient scavenging and toxin management. Properties that in pathogenic fungi have a role in virulence may have very different functionalities in the fungus’ natural habitat. Song et al. (2017) noted that hypothesized virulence factors, which enhance infection in truly host-associated fungi like *Candida* or *Histoplasma*, in black yeasts may respond adversely to proxies of host resistance, and thus comparable genes can be functionally different between fungal groups.

An important consequence of opportunism is that all strains have an equal chance to be inoculated into the host, and thus clinical strains are not necessarily different from environmental ones. Pathogenic adaptation is then less likely to occur because of absence of host-related transmission. However, after coincidental inoculation, some strains may be more prone to cause symptoms than others, and thus there may be a difference between clinical and environmental strains. Black yeast-like fungi mostly cause local, (sub) cutaneous infections. The host conditions of patients with brain abscesses acquired via the inhalative route are not well understood. For disseminated cases, recent data have shown (Lanternier et al., 2013) that these are often associated with host-inherited mutations in caspase recruitment domain-containing protein 9 (CARD9). This protein plays a role in the dectin pathway regulating innate immunity activating pro-inflammatory cytokines. Since most cases of disseminated disease in black yeast-like fungi were described prior to 2013, it is possible that the majority of these cases occurred in patients with CARD9 dysfunctional innate immunity. Several of the infection types of chaetothyrialean fungi show a certain degree of unexplained endemism, such as the prevalence of *Cladophialophora* brain abscess in the Indian subcontinent (Chakrabarti et al., 2016). Similarly, the majority of CARD9-associated black fungal cases is found in East Asia: China, Korea, Japan, although the etiologic agents have a global distribution. For an explanation of virulence of members of Chaetothyriales, we thus might expect answers from the interaction of the fungus with windows of opportunity associated with CARD9 regulation and human race.

To study the fungal side of this tripartite relationship, we sequenced the genomes and transcriptomes with different culture conditions of an *Exophiala spinifera* strain isolated from a patient with a *CARD9*-related disorder, and compared this with two environmental strains of the same species, and with other published genomes (Supplementary Table S1). We applied state-of-the-art technology, to quantify eventual differences between clinical and environmental strains with maximum precision and to describe the species’ intraspecific variability. A number of genomes of Chaetothyriales were already available, but these were sequenced with somewhat older techniques. One of the aims is therefore to describe the technical and biological variation in these datasets, and determine whether the new techniques provide a better answer to questions as formulated above.

## Materials and Methods

### Literature Search

Keywords “CARD9” and “fungal infection” were used in PubMed to search the English literature including research articles, reviews and case reports published until November 2019.

### Strains and Culture Conditions

Three strains of *E. spinifera* were analyzed (Table 1): BMU 08022 (**ESC1**, from a CARD9-deficient patient, Jiangsu, China), BMU00051 (**ESE1**, from bark, Shenzen, China), and BMU00047 (**ESE2,** from soil, Colombia). To prepare DNA for genome sequencing, mycelia were harvested from fresh cultures on Sabouraud Dextrose Agar (SDA), frozen in liquid nitrogen, and stored at −80°C until further processing. For RNA extraction, strains were inoculated in Sabouraud Dextrose Broth (SDB) and in Brain Heart Infusion Broth (BHI) (Das et al., 2010) and harvested after 20 h, centrifuged and frozen in liquid nitrogen. Liquid nitrogen was used to grind the samples for DNA/RNA extraction.

**TABLE 1:** Isolation data of strains sequenced in this study, and isolates included for comparison.

Strain ID	Species	Country	Source	GenBank Accession
BMU 08022	*E. spinifera*	China	Human skin (*CARD9* deficient)	JAAABF000000000
BMU 00051	*E. spinifera*	China	Bark	JAAABG000000000
BMU 00047	*E. spinifera*	Colombia	Soil	JAAABH000000000
CBS 899.68T	*E. spinifera*	United States	Human nasal granuloma	JYBY00000000
CBS 725.88T	*Exophiala oligosperma*	Germany	Human sphenoid abscess	JYCA01000000

### DNA and RNA Extraction

Genomic DNA was extracted using the MOBIO Power Microbial Maxi DNA Isolation kit (MoBio Laboratories, Carlsbad, CA, United States). DNA was concentrated by QUBIT (Invitrogen, Carlsbad, CA, United States), and an Agilent Bio Analyzer 2100 using a 1000 DNA Chip (Agilent Technologies, Palo Alto, CA, United States), and its quality was determined by electrophoresis on agarose gels. Total RNA was extracted from cultures ground in liquid nitrogen using TRIZOL reagent (Thermo Fisher Scientific, Waltham, MA, United States) according to the manufacturer’s instructions, followed by two phenol (pH 4.6)-chloroform-isoamyl alcohol (25:24:1) extraction steps and two chloroform extraction steps after the initial TRIZOL-chloroform phase separation. RNA pellets were dissolved in 88 μL of nuclease-free water and subjected to genomic DNA digestion with DNase (Qiagen, Hilden, Germany). RNA samples were then concentrated using RNA Clean & Concentrator (Zymo Research, Irvine, CA, United States). RNA quality was confirmed using an Agilent 2100 Bioanalyzer (Agilent). Two micrograms of total RNA per sample was used for cDNA library construction.

### cDNA Synthesis

The cDNA synthesis was performed using IMPRON II reverse transcriptase kit (Promega, Madison, WI, United States) with 1 μg of total RNA added according to manufacturer’s instructions. The efficiency of the cDNA synthesis process was assessed by PCR using the following reference genes: β-tubulin and ITS. The integrity of PCR amplification products was verified by electrophoresis on 1.2% agarose gels.

### PacBio Sequencing and Raw Assembly

The extracted DNA of three *E. spinifera* strains were sequenced on the Pacific Biosciences (Pacific Biosciences, Menlo Park, CA, United States) Single Molecule, Real-Time (SMRT) DNA sequencing technology (platform: RS II; chemistry: P6-C4). The raw reads were processed using the standard SMRT analysis pipeline v5.0.1 (Chin et al., 2013). The *de novo* assembly was carried out following CANU v1.4 assembly protocol (Koren et al., 2017) with QUIVER polishing (Chin et al., 2013). Assemblies were refined by manual curation. Pairwise alignments were carried out using BLASTN v2.6.0+ (Altschul et al., 1990). The draft assemblies were further improved with FINISHERSC (Lam et al., 2015) and polished with ARROW (Chin et al., 2013). For each genome assembly, contigs were examined and removed if redundant (i.e., aligning to any other contig in the same assembly with >90% identity). All contigs containing rDNA repeats were excluded from the above step.

### Illumina Sequencing, Read Mapping and Error Correction

**FIGURE 1:**
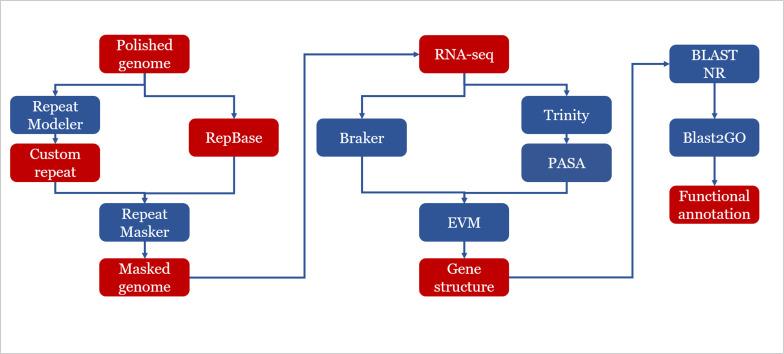
Genome assembly and annotation strategy used in this study. Assembly: the raw reads were processed using the standard SMRT analysis pipeline v5.0. The *de novo* assembly was carried out following CANU v1.4 assembly protocol with QUIVER polishing. Assemblies were refined by manual curation. Pairwise alignments were carried out using BLASTN v2.6.0+. The draft assemblies were further improved with FINISHERSC and polished with ARROW. Annotation: a hybrid strategy combining *ab initio* predictions and transcriptomic support (RNA-seq) was applied in gene prediction. Three *ab initio* gene finders, GENEMARK.HMM, FGENESH, and AUGUSTUS were used.

Our genome assembly strategy is summarized in Figure 1. In addition to the PACBIO sequencing, we also performed Illumina 150-bp paired-end sequencing for each strain. We examined the raw Illumina reads via FASTQC v0.11.5^1^ and performed adaptor-removal and quality-based trimming by TRIMMOMATIC v0.36 (Bolger et al., 2014). For each strain, the trimmed reads were mapped to the corresponding PACBIO assemblies by BWA 0.7.12 and using BWAMEM v0.7.16 (Li and Durbin, 2009). The resulting read alignments were subsequently processed by SAMTOOLS v1.6 (Li et al., 2009). On the basis of Illumina read alignments, we further performed error correction with PILON v1.22 (Walker et al., 2014) to generate final assemblies for downstream analysis. BUSCO v3 (Simão et al., 2015) was used to assess the completeness of the final assembled genomes.

### Repetitive Sequence Prediction and Masking

Repetitive elements were predicted using a *de novo* approach by applying REPEATMODELER v1.0.11^2^, which includes RECON v1.08 (Bao and Eddy, 2002) and REPEATSCOUT v1.0.5 (Price et al., 2005) to construct a strain-specific repeat library. To further classify repetitive sequences, BLAST v2.2.28 (Camacho et al., 2009) was used to search the repeat library against SWISS-PROT protein database. Sequences with similarities to known proteins were discarded from the repeat library. Repetitive sequences were masked with REPEATMASKER v4.0.7^2^ using the *de novo* constructed library.

### Annotation of rRNA, tRNA and Repeat Elements

The rRNA loci were predicted by using RNAMMER v1.2 (Lagesen et al., 2007) and tRNA by TRNASCAN-SE v2.0 (Lowe and Eddy, 1997). Repeat elements were identified using REPEATMASKER v4.0.6^2^ based on REPBASE LIBRARY 20160829. REPEATMASKER was run with options that skipped the low-complexity DNAs masking for the purpose of gene prediction.

### Gene Prediction and Functional Annotation

A hybrid strategy combining *ab initio* predictions and transcript alignments was applied in annotation of protein coding genes. Firstly, protein coding genes were predicted using BRAKER (Hoff et al., 2016), which was an *ab initio* approach combined with RNA-seq data. Then, RNA-seq was *de novo* assembled by using TRINITY (Grabherr et al., 2011). The assembled transcripts were aligned to the reference genome of corresponding strains by PASA pipeline (Haas et al., 2003). Finally, EVIDENCEMODELER (Haas et al., 2008) was used to combine *ab initio* predictions and transcript alignments into consensus gene structure.

Functional annotation of predicted proteins was done by BLAST (Camacho et al., 2009) similarity search of the predicted proteins against NR database with an e-value of 1e–05. Gene Ontology (GO) terms were retrieved by using Blast2go (Gotz et al., 2008) with default annotation parameters. To further improve the annotations, INTERPROSCAN v5.33-72.0 (Jones et al., 2014) was used to identify known protein domains. BLAST (Camacho et al., 2009) results of NR database and results of the INTERPROSCAN analysis were combined to gain a complete functional annotation of all predicted genes. Genes predicted to encode carbohydrate-active enzymes (CAZYMES) were identified with DBCAN2 (Zhang et al., 2018). Also the software SMURF (Khaldi et al., 2010) was used to predict secondary metabolite gene clusters in three genomes of *E. spinifera*.

### Comparison of Genome Assemblies

The synteny among three strains of *E. spinifera* was determined using MUMMER v3.23 (Delcher et al., 2003). We used NUCMER implemented in MUMMER v3.23 to align the genomes of the three strains of *E. spinifera*. In addition, the DNADIFF package in MUMMER v3.23 was used to generate coordinates for the differences among genomes of the three strains analyzed. The alignment statistics, SNPs, indels, inversions, etc., were also calculated from the results of MUMMER. We plotted these features circularly with CIRCOS v0.69.6 (Krzywinski et al., 2009).

### Identification of Homology Groups

For nuclear protein-coding genes, we used INPARANOID v4.1^3^ to identify gene homology across the three strains of *E. spinifera*. We calculated protein length distributions for each homologous group (1:1:1 association among strains) of the strains. The correlation of exon length and intron length of homologous groups (1:1 association between strains) were calculated by pairwise comparisons among three strains.

### Identification of Orthologous Genes

Orthologous groups were delimited using ORTHOFINDER v2.2.6 (Emms and Kelly, 2015), in which all predicted protein sequences were compared using a BLAST (Camacho et al., 2009) all-against-all search. The single-copy genes, duplicated genes, and strain-specific genes were extracted from the ORTHOFINDER output. To detect the core genome, specific and shared genes, the data was filtered based on the ORTHOFINDER output. The R package UPSETR (Conway et al., 2017) was used to construct statistics of orthologous groups among the three strains under study.

### Identification of Genes Under Positive Selection

Single-copy orthologous proteins were aligned by using CLUSTALO v1.2.4^4^. To obtain the alignments of codons, the corresponding nucleotide-sequence alignments were derived by substituting the respective coding sequences from the protein sequences. For each single-copy orthologous group, genes from the two environmental strains and their orthologous genes in the clinical strain formed sequence triplets. The test for the asymmetric evolution was constituted as a relative rate test between clinical strain and environmental strains on an unrooted tree. The statistical tests were conducted with a codon-based branch-site model using the CODEML program of PAMLv4.9 (Yang, 2007). We compared ω0 and ω1 between single-copy orthologous genes to detect differences in proportion of selected sites in the two clades. Likelihood ratio (LR) was used to test for significance. To do this, two models were applied to the data: model 1 (ω0 = ω1) constrains the two ω values to be equal for the three sequences, and model 2 (ω0 ≠ ω1) estimates the two ω values as free parameters. Collected maximum likelihood values ML1 and ML2 from the two models were used to calculate the LR, LR = 2 (lnML2−lnML1). LR is then compared against the χ^2^ distribution with one degree of freedom.

### Transcriptome Analysis

The raw reads were assessed for quality by FASTQC v0.11.5 and filtered to remove low-quality reads with TRIMMOMATIC v0.36 (Bolger et al., 2014). Filtered reads were mapped to the corresponding reference genome of three *E. spinifera* strains using STAR v2.5.3a (Dobin et al., 2013). The gene expression levels were calculated using FEATURECOUNTS v1.5.2 (Liao et al., 2014) and normalized based on the FPKM method. Differentially expressed genes were detected by R package DESEQ2 (Love et al., 2014). Differentially expressed genes were filtered by adjusted *p*-value < 0.05 and | log2 fold change| > 1. GO enrichment analysis was performed by R package TOPGO^5^. Only 1-1-1 orthologous genes were considered when detecting differentially expressed genes among different strains. For analysis of RNA-seq data, differentially expressed genes were detected by R Bioconductor package DESeq2 with the Benjamini-Hochberg adjusted *p*-value < 0.05 and fold change >2. Student’s *t*-tests were performed for the comparisons, and a *p-*value < 0.05 was considered to represent significance.

### Data Availability

All genomes and RNA-seq have been deposited in NCBI. The RNA-seq data can be accessed by PRJNA600825, the PACBIO assemblies (PRJNA600825), Illumina assemblies (PRJNA600036). The RNA-seq is available already (https://www.ncbi.nlm.nih.gov/sra/PRJNA600825).

## Results

### Literature Data on Black Fungi in CARD9 Patients

**FIGURE 2:**
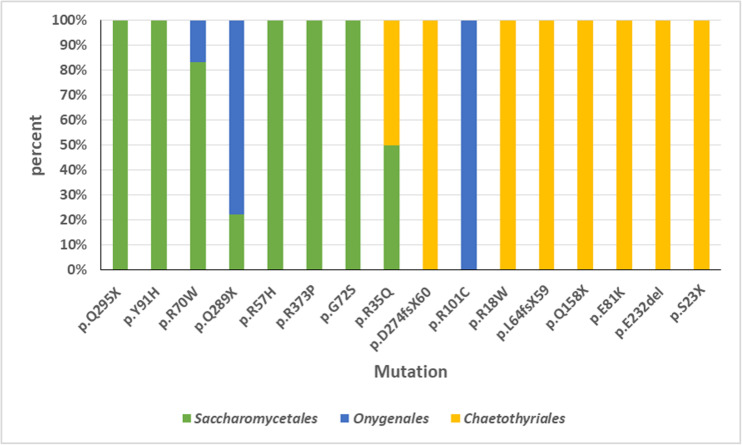
Overview of *CARD9* mutations in published cases of fungal infections in patients with CARD9 deficiencies. Sixteen different mutations have been observed. Three main groups can be recognized: ascomycetous yeasts (*Candida*: order Saccharomycetales), dermatophytes (order Onygenales), and black fungi (mainly order Chaetothyriales, single representatives of orders Pleosporales and Venturiales). A single proven case with a member of Mucorales has been published. Mutations are not randomly distributed in the three fungal groups.

**FIGURE 3:**
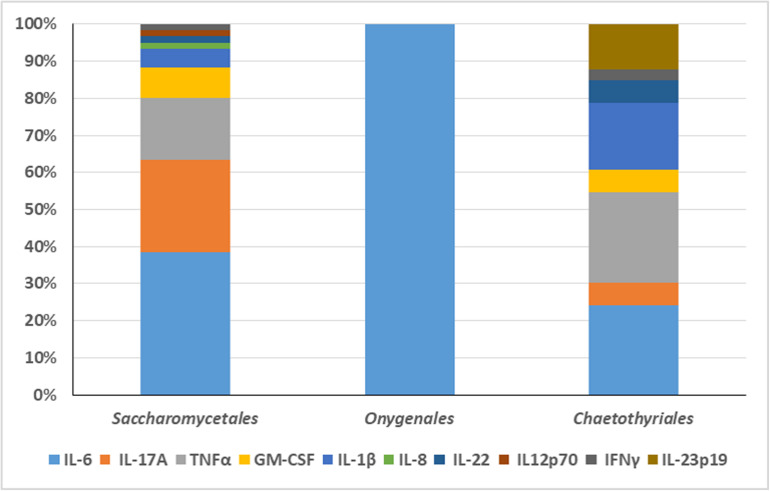
The relative absence of cytokines in the three fungal main groups is illustrated. The main cytokine impaired in *Candida* infection was IL-6. GM-CSF or TNFα were absent in seven cases of *Candida* infection. In Onygenales, IL-6 was the only deficient cytokine reported. Black fungi were associated with a relatively large panel of missing cytokines, particularly TNFα and IL-1β.

An overview was made of published cases of fungal infections in patients with CARD9 deficiencies (Table 2; Glocker et al., 2009; Drewniak et al., 2013; Lanternier et al., 2013, 2015a,b; Gavino et al., 2014, 2016; Drummond et al., 2015; Grumach et al., 2015; Herbst et al., 2015; Jachiet et al., 2015; Alves de Medeiros et al., 2016; Celmeli et al., 2016; Yan et al., 2016; Boudghene et al., 2017; Zhang et al., 2017; Arango-Franco et al., 2018; Cetinkaya et al., 2018; De Bruyne et al., 2018; Zhong et al., 2018; Huang et al., 2019). Sixteen different mutations have been observed. When cases are arranged according to phylogeny of the fungus, three main groups can be recognized: ascomycetous yeasts (*Candida*: order Saccharomycetales), dermatophytes (order Onygenales), and black fungi (mainly order Chaetothyriales, single representatives of orders Pleosporales and Venturiales). A single proven case with a member of Mucorales has been published. Mutations are not randomly distributed in the three fungal groups (Figure 2; *p* = 0.001). For example, p.Q295X is found in 13 cases of *Candida* infection, but was not found among cases by other fungi. The main cytokine impaired was IL-6 (43/50 cases). In Onygenales, this was the only deficient cytokine reported, while, in contrast, in seven cases of *Candida* infection GM-CSF or TNFα were absent instead. Black fungi were associated with a relatively large panel of missing cytokines, particularly TNFα and IL-1β. The relative absence of cytokines in the three fungal main groups is illustrated in Figure 3.

**TABLE 2:** Overview of published cases of fungal infection in patients with *CARD9* deficiency.

Origin	Outcome	Clinical	Species	Mutation type	Cytokine deficiency	References
**Saccharomycetales:**						
Iran	Died	CNS	*Candida albicans*	NR	NR	Glocker et al., 2009
Iran	Died	CNS, skin	*C. albicans*	NR	NR	Glocker et al., 2009
Iran	Died	CNS, mucosa	*C. albicans*	NR	NR	Glocker et al., 2009
Iran	Chronic	Mucosa	*C. albicans*	p.Q295X	TNFα, IL-17A	Glocker et al., 2009
Iran	Chronic	Skin	*C. albicans*	p.Q295X	TNFα, IL-17A	Glocker et al., 2009
Iran	Chronic	Oral, vagina, skin	*C. albicans*	p.Q295X	TNFα, IL-17A	Glocker et al., 2009
Iran	Chronic	Vagina, skin	*C. albicans*, dermatophyte	p.Q295X	TNFα, IL-17A	Glocker et al., 2009
Asia	Chronic	CNS	*Candida dubliniensis*	p.R373P/p.G72S	IL-6, IL-1β, IL-8, IL-17A	Drewniak et al., 2013
France	Chronic	CNS	*C. albicans*	p.Y91H	GM-CSF	Gavino et al., 2014
Turkey	Chronic	CNS	*C. albicans*	p.Q295X	IL-6, TNFα, IL-1β	Herbst et al., 2015
Turkey	Chronic	CNS	*C. albicans*	p.Q295X	NR	Celmeli et al., 2016
Turkey	Chronic	CNS	*C. albicans*	p.R70W	IL-6, TNFα	Lanternier et al., 2015a
Turkey	Chronic	CNS	*C. albicans*	p.R70W	IL-6, TNFα	Lanternier et al., 2015b
Iran	Chronic	CNS, sinus, digestive system	*Candida glabrata*	p.R35Q	IL-6, TNFα	Lanternier et al., 2015a
Morocco	Chronic	CNS	*C. albicans*	p.Q289X	NR	Lanternier et al., 2015b
Pakistan	Chronic	Digestive system	*C. albicans*	p.Q295X	IL-6, TNFα	Lanternier et al., 2015b
Canada	Chronic	CNS	*C. albicans*	p.Y91H	GM-CSF	Gavino et al., 2016
Canada	Chronic	CNS, spine	*C. albicans*	p.Y91H	GM-CSF	Gavino et al., 2016
United States	Chronic	CNS, bone	*C. albicans*	p.R57H	GM-CSF	Drummond et al., 2015
Turkey	Chronic	Mucosa	*C. albicans*	p.R70W	IL-6, TNFα, IFNγ, IL-1β, IL12p70	Alves de Medeiros et al., 2016
Turkey	Chronic	CNS, mucosa	*C. albicans*	NR	IL-6, GM-CSF, IL-22, IL-17A	Alves de Medeiros et al., 2016
Asia	Chronic	CNS	*C. dubliniensis*	G72S/R373P	NR	De Bruyne et al., 2018
El Salvador	Chronic	CNS, osteomyelitis	*C. albicans*	R57H/R57H	IL-6, IL-17A	De Bruyne et al., 2018
Canada	Chronic	CNS	*C. albicans*	Y91H/Y91H	IL-6, IL-17A	De Bruyne et al., 2018
Canada	Chronic	CNS	*C. albicans*	Y91H/c.-529T>C	IL-6, IL-17A	De Bruyne et al., 2018
Canada	Chronic	CNS, vertebral osteomyelitis	*C. albicans*	Y91H/c.-529T>C	IL-6, IL-17A	De Bruyne et al., 2018
**Saccharomycetales:**						
Canada	Chronic	CNS	NA	Y91H/c.-529T>C	IL-6, IL-17A	De Bruyne et al., 2018
Iran	Chronic	CNS	*Candida* sp.	Q295X/Q295X	IL-6, IL-17A	De Bruyne et al., 2018
Iran	Chronic	CNS	*Candida* sp.	NA	NA	De Bruyne et al., 2018
Iran	Chronic	CNS	*Candida* sp.	NA	NA	De Bruyne et al., 2018
Morocco	Chronic	CNS, papillary edema	*C. albicans*	Q289X/Q289X	IL-6, IL-17A	De Bruyne et al., 2018
Iran	Chronic	CNS	*C. glabrata*	R35Q/R35Q	IL-6, IL-17A	De Bruyne et al., 2018
Turkey	Chronic	CNS	*C. albicans*	R70W/R70W	IL-6, IL-17A	De Bruyne et al., 2018
Turkey	Chronic	CNS	*C. albicans*	R70W/R70W	IL-6, IL-17A	De Bruyne et al., 2018
Turkey	Chronic	CNS	*C. albicans*	NA	NA	De Bruyne et al., 2018
Turkey	Chronic	CNS	*C. albicans*	Q295X/Q295X	IL-6, IL-17A	De Bruyne et al., 2018
Turkey	Chronic	CNS	*C. albicans*	Q295X/Q295X	IL-6, IL-17A	De Bruyne et al., 2018
Turkey	Chronic	CNS	*C. albicans*	Q295X/Q295X	IL-6, IL-17A	De Bruyne et al., 2018
Turkey	Chronic	CNS	*C. albicans* (*Aspergillus* unconfirmed)	Q295X/Q295X	lL-6	De Bruyne et al., 2018
Europe	Chronic	CNS	*C. albicans, Aspergillus fumigatus*	Q295X/Q295X	IL-6	De Bruyne et al., 2018
**Onygenales:**						
Algeria	Chronic	Nail	*Trichophyton* sp.	p.Q289X	IL-6, IL-17A	Lanternier et al., 2013
Morocco	Chronic	Skin, nail, bone, lymph nodes	*Trichophyton rubrum*	p.R101C	NR	Lanternier et al., 2013
Morocco	Chronic	Head, nail	*Trichophyton* sp.	p.R101C	IL-6, IL-17A	Lanternier et al., 2013
Tunisia	Died	Skin, head, nail	*Trichophyton* sp.	p.Q289X	IL-6, IL-17A	Lanternier et al., 2013
Tunisia	Chronic	Head, nail	*T. rubrum*	p.Q289X	NR	Lanternier et al., 2013
Tunisia	Chronic	Skin, head, nail, lymph nodes	*T. rubrum, Trichophyton Violaceum*	p.Q289X	IL-6, IL-17A	Lanternier et al., 2013
Tunisia	Chronic	Skin, head, nail, lymph nodes	*T. rubrum, T. violaceum*	p.Q289X	IL-6, IL-17A	Lanternier et al., 2013
Egypt	Chronic	Skin, nail	*T. rubrum*	p.Q289X	IL-6, IL-17A	Jachiet et al., 2015
Brazil	Chronic	Skin	*Trichophyton mentagrophytes*	p.R101L	NR	Grumach et al., 2015
Algeria	Chronic	Skin, nail, head, lymph nodes, CNS	*T. rubrum*	p.Q289X	IL-6, IL-17A	Boudghene et al., 2017
Turkey	Chronic	Skin, nail, oral cavity, lymph nodes	*T. rubrum, T. violaceum, Trichophyton verrucosum, Malassezia*	p.R70W	IL-6, IL-17A	Alves de Medeiros et al., 2016
Algeria	Died	CNS	*T. violaceum*	NA (family member with Q289X)	IL-6, IL-17A	De Bruyne et al., 2018
**Chaetothyriales:**						
China	Chronic	Skin	*Phialophora verrucosa*	p.L64fsX59/p.Q158X	IL-6, TNFα, IL-1β, IL-23p19	Walker et al., 2014
China	Chronic	Skin	*P. verrucosa*	p.D274fsX60	IL-6, TNFα, IL-1β, IL-23p19	Walker et al., 2014
China	Chronic	Skin	*P. verrucosa*	p.D274fsX60	IL-6, TNFα, IL-1β, IL-23p19	Wang et al., 2014
China	Chronic	Skin	*P. verrucosa*	p.D274fsX60	IL-6, TNFα, IL-1β, IL-23p19	Zhang et al., 2018
China	Chronic	Skin, mucosa, sinus, CNS	*P. verrucosa*	p.R35Q/p.E81K	IL-6, TNFα	Zhang et al., 2018
Angola	Chronic	Liver, CNS	*Exophiala dermatitidis*	p.R18W	NR	Lanternier et al., 2015a
Iran	Chronic	Skin, bone, lung	*E. spinifera*	p.E232del	IL-6, TNFα, IL-1β, IFNγ, IL17, IL-22, GM-CSF	Lanternier et al., 2015b
China	Chronic	Skin	*E. spinifera*	p.S23X/p.D274fsX60	IL-6, TNFα, IL-1β, IL-17A, IL-22, GM-CSF	De Bruyne et al., 2018
Angola	Chronic	CNS	*E. dermatitidis*	R18W/R18W		De Bruyne et al., 2018
China	Chronic	Skin	*Periplaneta americana*	p.D274fsX60	IL-6, TNFα, IL-1β	Huang et al., 2019
**Venturiales:**						
China	NR	Skin	*Ochroconis musae*	p.D274fsX60	IL-6, TNFα, IL-1β, IL-17A, IL-22, GM-CSF	
**Pleosporales:**						
China	NR	Skin, mucosa	*Corynespora cassiicola*	p.L64fsX59/p.D274fsX60	IL-6, TNFα, IL-1β, IL-17A, IL-22, GM-CSF	Yan et al., 2016
China		Skin	*Corynespora cassiicola*	c.191–192InsTGCT, p.L64fsX59		Yan et al., 2016
Colombia		face	*Corynespora cassiicola*	c.23_29del; p.Asp8Alafs10		
				c.865C >T, p.Q289		Arango-Franco et al., 2018
**Mucorales:**						
China	Chronic	Skin	*Mucor irregularis*	c.692C>T/c.905_907delTCT	NR	Yan et al., 2016

### Genome Sequencing and Assembly

We sequenced the genomes of three strains: BMU 08022 (clinical strain from human skin, **ESC1**), BMU 00051 (environmental strain from bark, **ESE1**), and BMU 00047 (environmental strain from soil, **ESE2**) (Table 1) using a combination of long-read (PACBIO) and short-read (Illumina) sequencing technologies. The third generation sequencing is supplemented by the second generation sequencing, and combined with a variety of assembly software and parameter optimizations (Figure 1). For each genome, PACBIO sequencing provided more than 100 × coverage and Illumina reached 170× coverage. For strain ESC1, we obtained a draft genome assembly of 33,559,924 bp organized into nine contigs with an L90 of 7, and an N50 of 4.0 Mb. The genome of ESE1 was 32,380,025 bp in size and comprised seven contigs with an L90 of 7, and N50 of 4.9 Mb. In ESE2, the genome assembly of 32,696,644 bp contained nine contigs with an L90 of 7, and an N50 4.6 Mb. For all three strains, more than 94% of the Illumina reads were aligned to the draft genome assemblies in *post hoc* validation (Table 3). The results of genome assembly of the three strains reached sub-chromosome level.

**TABLE 3:** Genome assembly of analyzed strains of *Exophiala spinifera*, compared with deposited genomes of *E. spinifera* (CBS 899.68) and *E. oligosperma* (CBS 725.88).

	ESC1	ESE1	ESE2	CBS 899.68	CBS 725.88
Genome size (bp)	33,559,924	32,380,025	32,696,644	32,912,300	38,224,500
Number of contigs	9	7	9		
Number of scaffolds	8	7	7	28	143
L90	7	7	7		
N50 (bp)	4,048,587	4,872,376	4,635,272		
GC content	51.8%	51.9%	51.7%	51.7%	50.4%
CEGMA	236 (95.2%)	236 (95.2%)	237 (95.6%)		
BUSCOs	1,282 (97.5%)	1,276 (97.1%)	1,274 (96.9%)		
Number of genes	12,130	12,072	12,043	12,110	11,938
Number of proteins	12,131	12,074	12,043	12,049	13,234
Mean gene length (bp)	2,006	2,012	1,979		
Mean number of exons	2.84	2.92	2.87		
Genome coding	72.4%	75.0%	72.9%		
Coverage of InterPro	78.8%	77.6%	77.9%		
Coverage of GO	70.8%	70.0%	70.5%		
rRNA	18	28	21	16	4
tRNA	42	44	44	45	41
Genome coverage	103×	122×	110×	272×	240×
Sequencing technology	PacBio	PacBio	PacBio	Illumina	Illumina

*CEGMA, Core Eukaryotic Genes Mapping Approach; BUSCOs, Benchmarking Universal Single-Copy Orthologs.*

### Colinear Analysis

**FIGURE 4:**
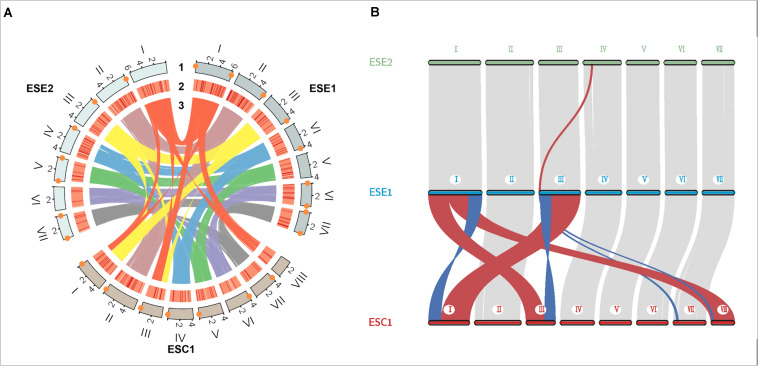
Genome colinear analysis for the three *E. spinifera* genomes. **(A)** Recombination is observed between clinical strain ESC1 and environmental strains ESE1 and ESE2 in scaffolds I and III. Track 1, genome position; track 2, gene density; track 3, sequence synteny. Orange diamonds: telomere sequences. **(B)** Location of sequence rearrangements in scaffolds I and III of ESC1. Red, stands for positive collinearity; Blue, reverse complementary.

We used whole-genome alignment to identify homologous and strain-specific segments among three *E. spinifera* strains. We identified a total of 172,269 and 170,807 SNPs for the clinical strain compared to the environmental strains BMU 00051 and BMU 00047, respectively (Table 4). However, the number of SNPs between the environmental strains was 362,264, which indicated that the genome of the clinical strain was intermediate between the two environmental strains from different continents. Likewise, the number of indels identified in the clinical strain was higher than compared with environmental strains (Table 4). There were 105,095 (ESE1) / 104,446 (ESE2) synonymous vs. 67,174 (ESE1) / 66,361 (ESE2) non-synonymous SNPs compared to clinical strain ESC1 (Table 4). We found substantial syntenic rearrangements in scaffolds I and III between ESC1 vs. ESE1 and ESC1 vs. ESE2, while in scaffolds VII and VIII of ESC1 syntenic rearrangements existed with scaffold III of ESE1 (Figure 4). Comparing the genomes of ESC1, ESE1, and ESE2, recombination was observed in scaffolds I and III between the clinical and environmental strains (Figure 4B). Scaffold I of ESC1 is equal to part of scaffolds I and III of the environmental strains, and scaffold III of ESC1 is equal to the remainders of scaffolds I and III of the environmental strains. This is shown in more detail in Figure 4, where scaffold VIII of ESC1 is shown to be comparable to part of scaffold I of the environmental strains, and two areas proved to be inverted between clinical and environmental strains.

**TABLE 4:** Comparison of single nucleotide polymorphisms of clinical vs. environmental strains.

	ESC1 vs. ESE1	ESC1 vs. ESE2
Synonymous SNP	105,095	104,446
Non-synonymous SNP	67,174	66,361
Frameshift deletion	1,231	1,152
Frameshift insertion	1,124	1,133
Non-frameshift insertion	965	962
Non-frameshift deletion	939	909
Stopgain	701	849
Stoploss	153	150

### Gene Content and Functional Annotation

**FIGURE 5:**
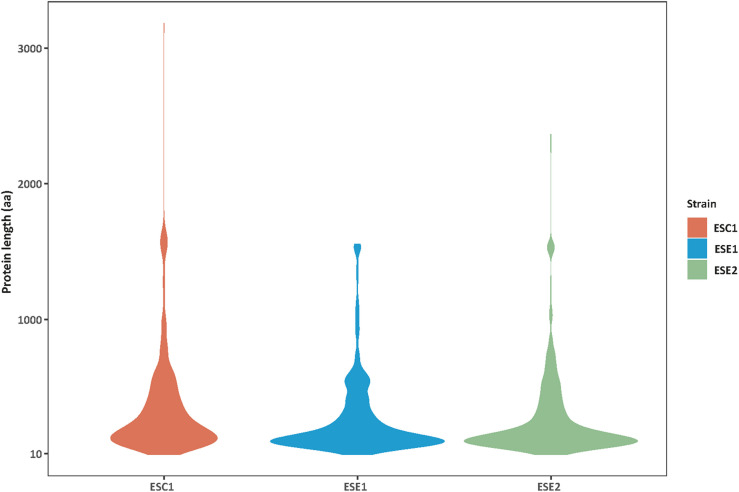
Distribution variability of specific protein length among three strains. The protein lengths (*Y*-axis) in strains analyzed (*n* = *X*-axis) showing an intraspecific variability. Greater differences were found between clinical strain ESC1 and environmental strains ESE1 and ESE2, while the protein length distribution of the two environmental strains was more consistent.

**FIGURE 6:**
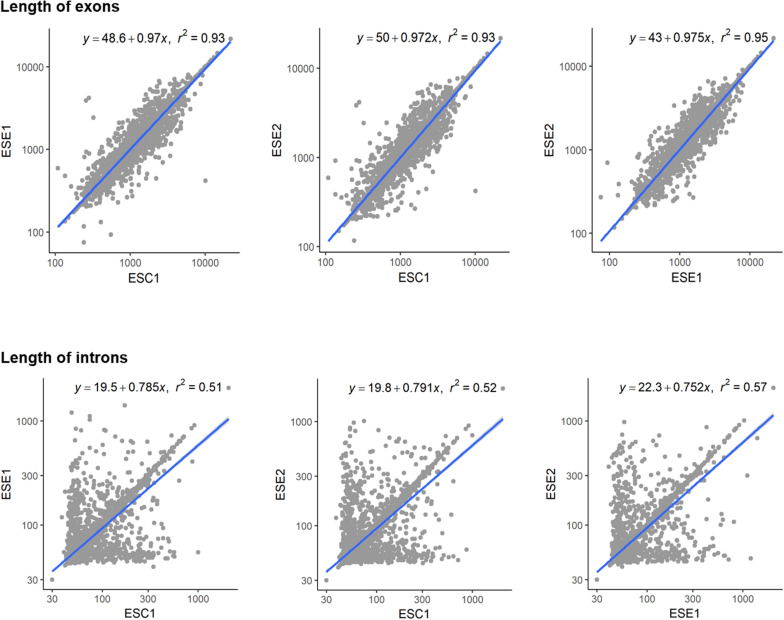
Length variations of introns and exons. The blue lines show trends in exon length distribution, the slopes being different between exons and intron. Positive and negative correlations are shown. Homologous introns were weakly correlated, with significant length variations.

Genome functional annotation was performed with INTERPROSCAN, resulting in over 59% of genes being protein-coding genes annotated with GO term. A hybrid strategy combining *ab initio* predictions and transcriptomic support (RNA-seq) was applied in gene prediction. Annotation of the strains yielded 12,131 protein-coding genes for *E. spinifera* ESC1, 12,074 protein-coding genes for ESE1 and 12,043 for ESE2 (Table 3). A total of 9,887 genes represented the core genome (Supplementary Figure S1). The environmental strains shared more genes (336) than compared with clinical strain ESC1. The clinical strain had 647 specific gene clusters, included transporter, and binding protein. The list of the respective functional annotations is available in Supplementary Table S2. Plotting the protein lengths of the three strains, considerable differences were noted (Figure 5): the distribution pattern of strain-specific genes length varied from clinical strain ESC1 to environmental strain, and ESC1 deviated by having more genes. Single-copy orthologous genes were compared to detect differences in proportion of selected sites, with LR as test of significance. In contrast, homologous introns were weakly correlated between ESC1 and environmental strain (ESE1 and ESE2), with significant length variations, while correlation were present between environmental strains (*r*^2^ = 0.57, higher than the other two groups) (Figure 6).

### Secondary Metabolite Clusters

**FIGURE 7:**
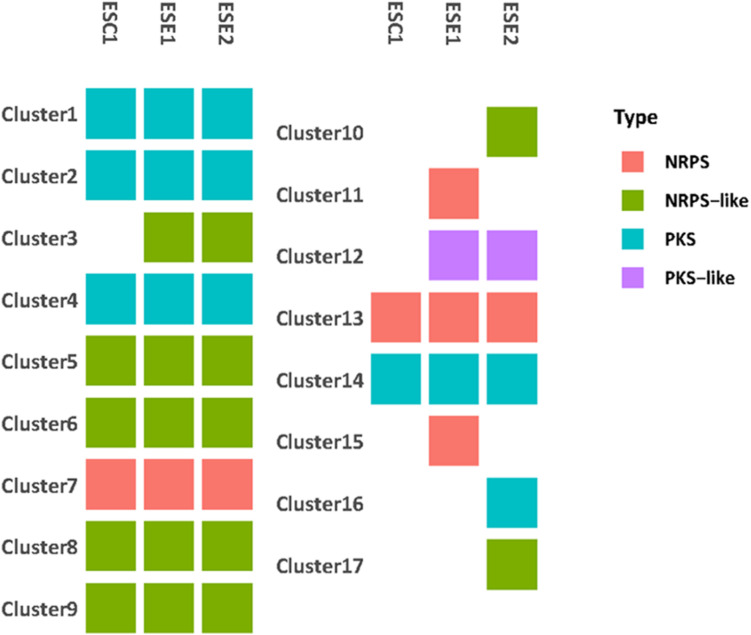
Genome comparison of secondary metabolite clusters of three strains. Seventeen gene clusters were involved in the production of secondary metabolites. Environmental strains ESE1 and ESE2 are predicted to have more secondary metabolite clusters than the clinical strain ESC1.Clusters 1, 2, 4, 14, and 16 are PKS-related clusters. Cluster 16 is present in environmental strain ESE2 only.

We identified a total of 9,329 homologous groups at the gene level (1:1:1 association among three strains). In the assumption that the core genomes with basic metabolic functions have similar biological functions, we focused on secondary metabolites. Seventeen gene clusters were involved in the production of secondary metabolites (Figure 7). Clusters 1, 2, 4, 14, and 16 are PKS-related clusters. Cluster 16 is present in environmental strain ESE2 only. The cluster contains the single-copy gene benzoate 4-monooxygenase cytochrome P450, which catalyzes the benzoate degradation step in the toluene catabolic pathway (Table 5).

**TABLE 5:** Function of specific secondary metabolic gene clusters.

Cluster ID	Strain	Backbone
Cluster 3	ESE1 and ESE2	L-aminoadipate-semialdehyde dehydrogenase
Cluster 10	ESE2	Putative acyl-coenzyme A synthetase
Cluster 11	ESE1	Nonribosomal peptide synthase Pes1
Cluster 12	ESE1 and ESE2	Beta-ketoacyl-acyl-carrier-protein synthase II
Cluster 15	ESE1	Nonribosomal peptide synthase GliP
Cluster 16	ESE2	Polyketide synthase, putative
Cluster 17	ESE2	NRPS-like enzyme

### RNA-seq

**FIGURE 8:**
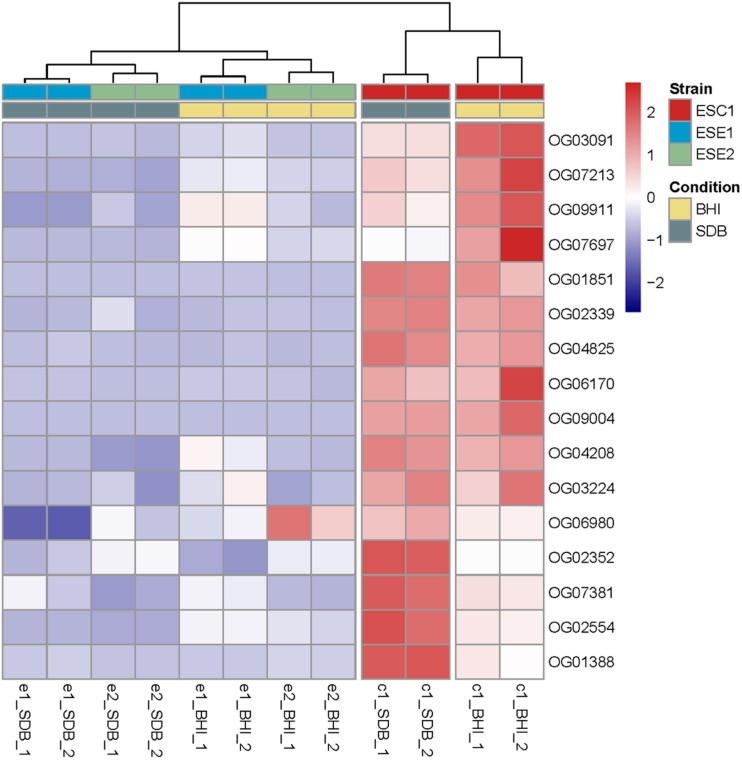
Comparison of differentially expressed genes upon incubation in SDB and BHI broth. Clustering of deviations from zero and shown in a dendrogram by complete linkage hierarchical clustering using Euclidean distance. All tests are shown in duplicate. The results demonstrated that ESC1 revealed significant differences in response to the two media in 16 genes, while the environmental strains ESE1 and ESE2 remained almost indifferent.

Sequence clustering at the mRNA level was used to compare the three strains to identify homologous groups among the three strains. For all three strains, poly (A)-enriched, strand-specific RNA-seq from two cultivation conditions (SDB and BHI broth) with different culture degrees was performed. Comparison of differentially expressed genes under different culture conditions demonstrated that ESC1 revealed significant differences in response to the two media in 16 single-copy genes, while the environmental strains ESE1 and ESE2 remained almost indifferent (Figure 8 and Supplementary Table S3).

There were 48 unique genes (22 in BHI media and 26 genes in SDB media; Table 6) involved in metabolism and transcriptional regulation differentially expressed exclusively in the clinical strain compared to the two environmental strains, including 12 genes without functional annotation. Supplementary Table S4 displays the functional gene content (name, IPR families, KOG, and GO) and respective expression levels in each scenery/media, to enable the visualization/filters of this data. The table includes the gene ID, description, IPR numbers and the GO information. Differentially expressed genes between clinical isolate and environmental isolates under BHI culture are listed in Supplementary Table S5. There were 637 up-regulated genes between ESC1 and ESE1, and 659 up-regulated genes between ESC1 and ESE2. These specific upregulated genes in the clinical strain mainly act in transmembrane transport, translational elongation, ribosome biogenesis, and some other biosynthetic processes (GO enrichment results are summarized in Supplementary Table S6). GO analysis showed that the gene annotations were mainly in three categories: biological processes (BP), molecular function (MF), and cell component (CC). GO categories among three strains showed a similar enrichment distribution (Supplementary Figure S2). However, GO annotations of genes unique to each strain were not identical in BP and MF ontologies. In the biological process, the differentially expressed genes were mainly enriched in response to cellular process and metabolic process. In the MF, differentially expressed genes were mainly related to binding and catalytic activity (Supplementary Figure S3).

**TABLE 6:** Unique genes differentially expressed exclusively in the clinical strain.

Gene Ontology	Total genes	Media
NA (NA)	10	SDB
integral component of membrane (GO:0016021)	6	SDB
NA (NA)	2	BHI
nucleus (GO:0005634)	2	BHI
oxidation-reduction process (GO:0055114)	2	BHI
oxidoreductase activity (GO:0016491)	2	BHI
zinc ion binding (GO:0008270)	2	BHI
carbon-sulfur lyase activity (GO:0016846)	2	SDB
DNA-binding transcription factor activity (GO:0003700)	2	SDB
GTP binding (GO:0005525)	2	SDB
ligase activity (GO:0016874)	2	SDB
methylation (GO:0032259)	2	SDB
methyltransferase activity (GO:0008168)	2	SDB
phosphopantetheine binding (GO:0031177)	2	SDB
regulation of transcription, DNA-templated (GO:0006355)	2	SDB
transcription factor complex (GO:0005667)	2	SDB
transmembrane transport (GO:0055085)	2	SDB
catalytic activity (GO:0003824)	1	BHI
integral component of membrane (GO:0016021)	1	BHI
protein transport (GO:0015031)	1	BHI
ATP binding (GO:0005524)	1	SDB
cytoplasm (GO:0005737)	1	SDB
DNA-binding transcription factor activity, RNA polymerase II-specific (GO:0000981)	1	SDB
nucleus (GO:0005634)	1	SDB
regulation of transcription by RNA polymerase II (GO:0006357)	1	SDB
ribosome (GO:0005840)	1	SDB
RNA binding (GO:0003723)	1	SDB
transferase activity (GO:0016740)	1	SDB
zinc ion binding (GO:0008270)	1	SDB

The correlation between differential expression genes and positive selection genes are listed in Supplementary Table S7. There were 53 positively selected genes that were also up-regulated between ESC1 and ESE1 in BHI culture. Comparing ESC1 and ESE2 under BHI conditions, 53 positively selected genes were also up-regulated in ESC1. A total of 29 positively selected genes were up-regulated in ESC1 when compared with both ESE1 and ESE2 in BHI broth. These genes were involved in positive regulation of translation, regulation of translational termination, and ATP synthesis coupled proton transport.

### Genes Under Positive Selection

Models 1 (ω0 = ω1) and 2 (ω0 ≠ ω1) were used to estimate the ω values as free parameters, with LRs as parameters of significance. A total of 29 genes yielded significant values indicating positive selection (*p* ≤ 0.0001) (Supplementary Table S8). These genes were subjected to GO analysis and were mainly enriched for BP, including cellular response, positive regulation of phosphorylation and transferase activity. Some replication- and transcription-associated genes and several structural protein genes were also discovered under positive selection (Supplementary Table S9).

## Discussion

Black yeasts are generally considered to be opportunists; consequently, they should lack any specialized adaptation to the vertebrate host (Seyedmousavi et al., 2018). Infection is coincidentally promoted by factors that enhance survival in the environmental niche of the fungus. Such factors must be present in chaetothyrialean black fungi, given the relatively large number of species reported from infections in humans and in cold-blooded animals. The Chaetothyriales are particularly over-represented in patients with inherited deficiencies in caspase recruitment domain-containing signaling protein 9 (CARD9), a disorder impairing the dectin pathway of innate immunity. This protein enhances pattern recognition receptors to induce NF-κB and MAPK activation, leading to a cytokine cascade. Malfunctioning of the protein decreases protection against infection by larger microbes such as parasites and especially fungi. It has been puzzling why the patients, theoretically being susceptible to any fungal infection, usually carry only a single species. Reviewing the 77 fungal cases published to date (Table 1), a possible explanation might be found in the fact that this protection deficiency appears to be highly specific. Only three groups of fungi are prevalent: *Candida* spp. (order Saccharomycetales), dermatophytes (order Onygenales) and black fungi of the order Chaetothyriales. A single case of *Mucor irregularis* has been published. This is the only species of *Mucor* that is known to cause chronic infections in apparently healthy individuals (Lu et al., 2013), other Mucorales being acute in compromised patients. As noted by Vaezi et al. (2018), there is a regional bias in CARD9 deficiency cases in that the great majority of cases by *Candida* and dermatophytes are from northern Africa, Turkey, and Iran, while chaetothyrialean cases are particularly encountered in East Asia. Notably, severe and chronic black fungal infections are well-known in China (de Hoog et al., 2020). However, most cases of disseminated fungal infection in “healthy” patients were published before CARD9 deficiency was discovered, thus the possibility is not excluded that more patients had a hidden inherited immune disorder. Remarkably, common opportunists such as *Aspergillus* or *Fusarium* are nearly or completely lacking. The over-representation of chaetothyrialean black fungi in this susceptible patient population is also striking because such infections are rare in other patient cohorts, in healthy as well as in otherwise immunocompromised hosts. In black fungi, i.e., members of Chaetothyriales, Pleosporales, and Venturiales, 40% of the mutations were heterozygous. Mutation types tend to be different between the groups of yeasts, dermatophytes, and black fungi (Figure 2): black fungi responded to other mutations than yeasts and dermatophytes. Also the affected cytokines differed between groups.

From these data it is obvious that black fungi, and particularly those belonging to the order Chaetothyriales, respond in a rather specific manner to impairment of the human immune system. Comparing the three main groups (yeasts, dermatophytes, black fungi) with respect to their response to cytokines, quite remarkably, the patients with chronic dermatophyte infections all were reported to have IL-6 deficiency alone, while in black fungi TNFα and often also IL-17A were impaired (Figure 3). In *Candida*, a more variable picture was observed, but IL-6 deficiency was observed in 23 out of 31 cases. IL-6 is a proinflammatory cytokine controlling T-cell differentiation, particularly Th17 and regulatory T cells (Zhao et al., 2012). These conclusions should however be taken with some care, as cytokine measurements were not consistent between publications.

The data are nevertheless sufficient to surmise that black fungal infections, particularly of members of Chaetothyriales, are not randomly occurring in susceptible patients with impaired immunity in general, but respond specifically to the conditions provided by CARD9 impairment. Because of their pronounced ability to decompose monoaromatic toxins, black yeasts of this order are enriched in the domestic environment (Moreno et al., 2018), and therefore close vicinity to humans is probable. Many species are oligotrophic and have efficient nutrient scavenging systems. Toxin management has been hypothesized to enhance survival strategies in the environment (Gostincar et al., 2018), and industrial pollution by monoaromates might promote growth of these fungi near humans. In this scenario, take-up by susceptible individuals followed by successful infection, may explain their high frequency in CARD9 patients. The cytochrome P-450 gene family has been suggested as a possible factor explaining fungal neurotropism, but our clinical strain lacked PKS cluster 16 which is present in one of the environmental strains (Figure 5 and Table 4) and thus lacks benzoate 4-monooxygenase, an important gene in hydrocarbon catabolism.

Over the past decade, advances in Next Generation Sequencing (NGS) technologies and decreasing sequencing costs allowed an increase in the number of sequenced genomes, with better quality. Long-read sequencing technologies have contributed greatly to comparative genomics among species and can also be applied to study genomics within a species (Kim et al., 2019). The long-read sequencing method, PACBIO, has allowed the ability to obtain complete and accurate sequences. Genome data of our strains are listed in Table 2, showing a range of intraspecific variability of 863,280 bp and 87 genes. These genomes were sequenced by a combined Illumina and PACBIO strategy. We identify a relatively large core genome of 9,887 genes from our three genomes. The two environmental strains shared more genes (336) than a comparison with clinical strain ESC1. The clinical strain had 647 specific gene clusters. Comparing the genomes of *E. spinifera* sequenced to date, the older Illumina genomes were within the range of the above established species variance. Similarly, comparable numbers of genes were found, suggesting that published genomes are reliable, despite the fact that the number of scaffolds in the Illumina-only genomes was considerably higher.

In our study, the utility of long read sequencing is the possibility to investigate complex fungal genomes and characterize genomic variation. Despite surmised opportunism in black yeasts, our clinical strain of *E. spinifera*, derived from a CARD9-deficient patient, differed significantly from two environmental strains which originated from different continents. In the environmental/clinical comparison, rearrangements were observed in scaffolds I and III (Figure 4), including two inversions, and ESC1 having an extra scaffold VIII (Figure 4). The rearrangements in this genome area are significant (Figure 4B). In contrast, clinical strain ESC1 was intermediate between the two environmental strains in numbers of SNPs and Indels. This suggests that the genomic rearrangements were a saltational event in an otherwise homogeneous population, as was noted in *Candida* after passage through a murine host (Forche et al., 2009). If, in contrast to *Candida, E. spinifera* is an opportunist residing in a non-human habitat, no transmission takes place after infection, and thus mutations are unlikely to be maintained in the population (De Hoog et al., 2018). The possibility cannot be excluded that the rearrangements were acquired during presence in the CARD9-deficient patient. Whether or not these may contribute to increased virulence of the species in an evolutionary step, as suggested by Casadevall (2007), or if they are lost as supposed by De Hoog et al. (2018), requires study of a larger set of genomes and understanding of natural variability of *E. spinifera* in a wider array of habitats.

*E. spinifera* shows variable responses with hemolysis (Song et al., 2017). Although these authors noted that this ability did not match with the division environmental/clinical, the possibility is not excluded that hemolytic strains have an enhanced invasive potency for vertebrate hosts. We registered up- or down-regulation of single-copy proteins in response to SDB vs. BHI, and noted that ESC1 showed significant differences with the two media in 16 single-copy genes, while the environmental strains remained almost indifferent; *in vitro*, ESC1 and ESE1 showed hemolysis, while ESE2 was negative. Most of the single-copy genes upregulated with BHI were transporters in KOG classification. Moreover, there were 48 unique genes differentially expressed exclusively to the clinical strain in two different media, including genes from various metabolic processes and transcriptional regulation. Up-regulated genes in clinical strain in GO enrichment were mainly involved in transmembrane transport, biosynthetic process, and metabolic process.

In this study, significant genomic rearrangements between strains of the same species were demonstrated. Two environmental strains from different continents were largely identical, whereas a clinical strain from a CARD9-deficient patient was different and deviated in the number of genes, even though all strains were phenotypically similar (Song et al., 2017). In general, clinical and environmental strains of a single opportunistic fungus are taken to have comparable infectious abilities, because neither of them is equipped with specialized virulence factors. However, the observed quantitative genetic variability of strains of the single species *E. spinifera* possibly is associated with a differential chance to cause infection in susceptible patient populations; differences may be small but clinically relevant. We compared our NGS approach with older Illumina techniques, which showed some minor flaws e.g., in the number of scaffolds, but for quantitative description of the genome these data proved to be sufficient. The power of long reads obtained by PACBIO sequencing is to enable assembly of a complete genome sequence, which disclosed significant genomic rearrangements. Main improvement of PACBIO data are expected in qualitative studies of specific gene functions.

## Data Availability Statement

The datasets generated for this study can be found in the NCBI. The RNA-seq data can be accessed by PRJNA600825, the PacBio assemblies (PRJNA600825), Illumina assemblies (PRJNA600036). The RNA-seq is available already (https://www.ncbi.nlm.nih.gov/sra/PRJNA600825).

## Author Contributions

RL and GS designed the experiments and supervised the data analysis. YS performed the experiments and wrote the relevant portions of the manuscript. MD and EY analyzed the data. NM and VV provided the technical support. All authors discussed the results and commented on the manuscript.

## Conflict of Interest

The authors declare that the research was conducted in the absence of any commercial or financial relationships that could be construed as a potential conflict of interest.

## Footnotes

1 http://www.bioinformatics.babraham.ac.uk/

2 http://www.repeatmasker.org

3 http://inparanoid.sbc.su.se/

4 http://www.clustal.org/

5 https://bioconductor.org/

## References

[B1] Altschul S. F. Gish W. Miller W. Myers E. W. Lipman D. J. (1990). Basic local alignment search tool. *J. Mol. Biol.* 215 403–410. 10.1016/S0022-2836(05)80360-2 2231712

[B2] Alves de Medeiros A. K. Lodewick E. Bogaert D. J. A. Haerynck F. Van Daele S. Lambrecht B. (2016). Chronic and invasive fungal infections in a family with CARD9 deficiency. *J. Clin. Immunol.* 36 204–209. 10.1007/s10875-016-0255-8 26961233

[B3] Arango-Franco C. A. Moncada-Vélez M. Beltrán C. P. Berrío I. Mogollón C. Restrepo A. (2018). Early-onset invasive infection due to *Corynespora cassiicola* associated with compound heterozygous CARD9 mutations in a Colombian patient. *J. Clin. Immunol.* 38 794–803. 10.1007/s10875-018-0549-0 30264381

[B4] Bao Z. Eddy S. R. (2002). Automated de novo identification of repeat sequence families in sequenced genomes. *Genome Res.* 12 1269–1276. 10.1101/gr.88502 12176934 PMC186642

[B5] Bolger A. M. Lohse M. Usadel B. (2014). Trimmomatic: a flexible trimmer for Illumina sequence data. *Bioinformatics* 30 2114–2120. 10.1093/bioinformatics/btu170 24695404 PMC4103590

[B6] Boudghene O. Amrani N. Boudghéne Stambouli K. Bouali F. (2017). Dermatophytic disease with deficit in CARD9: a new case with a brain impairment. *J. Mycol. Med.* 27 250–253. 10.1016/j.mycmed.2017.01.001 28391957

[B7] Camacho C. Coulouris G. Avagyan V. Ma N. Papadopoulos J. Bealer K. (2009). BLAST+: architecture and applications. *BMC Bioinform.* 10:421. 10.1186/1471-2105-10-421 20003500 PMC2803857

[B8] Casadevall A. (2007). Determinants of virulence in the pathogenic fungi. *Fungal. Biol. Rev.* 21 130–132. 10.1016/j.fbr.2007.02.007 19513186 PMC2693381

[B9] Celmeli F. Oztoprak N. Turkkahraman D. Seyman D. Mutlu E. Frede N. (2016). Successful granulocyte colony-stimulating factor treatment of relapsing *Candida albicans* meningoencephalitis caused by CARD9 deficiency. *Pediatr. Infect. Dis.* 35 428–431. 10.1097/INF.0000000000001028 26658378

[B10] Cetinkaya P. G. Ayvaz D. C. Karaatmaca B. Gocmen R. Söylemezoðlu F. Bainter W. (2018). A young girl with severe cerebral fungal infection due to card 9 deficiency. *Clin. Immunol.* 191 21–26. 10.1016/j.clim.2018.01.002 29307770

[B11] Chakrabarti A. Kaur H. Rudramurthy S. M. Appannanavar S. B. Patel A. Mukherjee K. K. (2016). Brain abscess due to *Cladophialophora bantiana*: a review of 124 cases. *Med. Mycol.* 54 111–119. 10.1093/mmy/myv091 26483430

[B12] Chin C. S. Alexander D. H. Marks P. Klammer A. A. Drake J. Heiner C. (2013). Nonhybrid, finished microbial genome assemblies from long-read SMRT sequencing data. *Nat. Methods.* 10 563–569. 10.1038/nmeth.2474 23644548

[B13] Conway J. R. Lex A. Gehlenborg N. (2017). UpSetR: an R package for the visualization of intersecting sets and their properties. *Bioinformatics* 33 2938–2940. 10.1093/bioinformatics/btx364 28645171 PMC5870712

[B14] Das S. Sharma S. Kar S. Sahu S. K. Samal B. Mallick A. (2010). Is inclusion of Sabouraud dextrose agar essential for the laboratory diagnosis of fungal keratitis? *Indian J. Ophthalmol.* 58 281–286. 10.4103/0301-4738.64122 20534916 PMC2907027

[B15] De Bruyne M. Hoste L. Bogaert D. J. Van den Bossche L. Tavernier S. J. Parthoens E. (2018). A CARD9 founder mutation disrupts NF-κB signaling by inhibiting BCL10 and MALT1 recruitment and signalosome formation. *Front. Immunol.* 9:2366. 10.3389/fimmu.2018.02366 30429846 PMC6220056

[B16] De Hoog G. S. Ahmed S. A. Danesi P. Guillot J. Gräser Y. (2018). “Distribution of pathogens and outbreak fungi in the fungal Kingdom,” in *Emerging and Epizootic Fungal Infections In Animals*, ed. Seyedmousavi S. (Cham: Springer), 3–16.

[B17] de Hoog G. S. Guarro J. Gené J. Ahmed S. Al-Hatmi A. M. S. Figueras M. J. (2020). *Atlas Of Clinical Fungi*, 4th Edn, Utrecht: Westerdijk Fungal Biodiversity Institute.

[B18] Delcher A. L. Salzberg S. L. Phillippy A. M. (2003). Using MUMmer to identify similar regions in large sequence sets. *Curr. Protoc. Bioinform. Chapt.* 10:Unit1013. 10.1002/0471250953.bi1003s00 18428693

[B19] Dobin A. Davis C. A. Schlesinger F. Drenkow J. Zaleski C. Jha S. (2013). STAR: ultrafast universal RNA-seq aligner. *Bioinformatics* 29 15–21. 10.1093/bioinformatics/bts635 23104886 PMC3530905

[B20] Drewniak A. Gazendam R. P. Tool A. T. J. van Houdt M. Jansen M. H. van Hamme J. L. (2013). Invasive fungal infection and impaired neutrophil killing in human CARD9 deficiency. *Blood* 121 2385–2392. 10.1182/blood-2012-08-450551 23335372

[B21] Drummond R. A. Collar A. L. Swamydas M. Rodriguez C. A. Lim J. K. Mendez L. M. (2015). CARD9-dependent neutrophil recruitment protects against fungal invasion of the central nervous system. *PLoS. Pathog.* 11:e1005293. 10.1371/journal.ppat.1005293 26679537 PMC4683065

[B22] Emms D. M. Kelly S. (2015). OrthoFinder: solving fundamental biases in whole genome comparisons dramatically improves orthogroup inference accuracy. *Genome Biol.* 16 157–157. 10.1186/s13059-015-0721-2 26243257 PMC4531804

[B23] Forche A. Magee P. T. Selmecki A. Berman J. May G. (2009). Evolution in *Candida albicans* populations during a single passage through a mouse host. *Genetics* 182 799–811. 10.1534/genetics.109.103325 19414562 PMC2710160

[B24] Gavino C. Cotter A. Lichtenstein D. Lejtenyi D. Fortin C. Legault C. (2014). CARD9 deficiency and spontaneous central nervous system candidiasis: complete clinical remission with GM-CSF therapy. *Clin. Infect. Dis.* 59 81–84. 10.1093/cid/ciu215 24704721 PMC4305130

[B25] Gavino C. Hamel N. Zeng J. B. Legault C. Guiot M.-C. Chankowsky J. (2016). Impaired RASGRF1/ERK-mediated GM-CSF response characterizes CARD9 deficiency in French-Canadians. *J. Allergy Clin. Immunol.* 137 1178–1188. 10.1016/j.jaci.2015.09.016 26521038

[B26] Glocker E.-O. Hennigs A. Nabavi M. Schäffer A. A. Woellner C. Salzer U. (2009). A homozygous CARD9 mutation in a family with susceptibility to fungal infections. *N. Engl. J. Med.* 361 1727–1735. 10.1056/NEJMoa0810719 19864672 PMC2793117

[B27] Gostincar C. Stajich J. E. Zupancic J. Zalar P. Gunde-Cimerman N. (2018). Genomic evidence for intraspecific hybridization in a clonal and extremely halotolerant yeast. *BMC Genom.* 19:364. 10.1186/s12864-018-4751-5 29764372 PMC5952469

[B28] Gotz S. García-Gómez J. M. Terol J. Williams T. D. Nagaraj S. H. Nueda M. J. (2008). High-throughput functional annotation and data mining with the Blast2GO suite. *Nucleic Acids Res.* 36 3420–3435. 10.1093/nar/gkn176 18445632 PMC2425479

[B29] Grabherr M. G. Haas B. J. Yassour M. Levin J. Z. Thompson D. A. Amit I. (2011). Full-length transcriptome assembly from RNA-Seq data without a reference genome. *Nat. Biotechnol.* 29 644–652. 10.1038/nbt.1883 21572440 PMC3571712

[B30] Grumach A. S. de Queiroz-Telles F. Migaud M. Lanternier F. Filho N. R. Palma S. M. U. (2015). A homozygous CARD9 mutation in a Brazilian patient with deep dermatophytosis. *J. Clin. Immunol.* 35 486–490. 10.1007/s10875-015-0170-4 26044242

[B31] Haas B. J. Delcher A. L. Mount S. M. Wortman J. R. Smith R. K. Jr. Hannick L. I. (2003). Improving the *Arabidopsis* genome annotation using maximal transcript alignment assemblies. *Nucleic Acids. Res.* 31 5654–5666. 10.1093/nar/gkg770 14500829 PMC206470

[B32] Haas B. J. Salzberg S. L. Zhu W. Pertea M. Allen J. E. Orvis J. (2008). Automated eukaryotic gene structure annotation using EVidenceModeler and the program to assemble spliced alignments. *Genome Biol.* 9:R7. 10.1186/gb-2008-9-1-r7 18190707 PMC2395244

[B33] Herbst M. Gazendam R. Reimnitz D. Sawalle-Belohradsky J. Groll A. Schlegel P.-G. (2015). Chronic *Candida albicans* meningitis in a 4-year-old girl with a homozygous mutation in the CARD9 gene (Q295X). *Pediatr. Infect. Dis. J.* 34 999–1002. 10.1097/INF.0000000000000736 25933095

[B34] Hoff K. J. Lange S. Lomsadze A. Borodovsky M. Stanke M. (2016). BRAKER1: unsupervised RNA-seq-based genome annotation with GeneMark-ET and AUGUSTUS. *Bioinformatics* 32 767–769. 10.1093/bioinformatics/btv661 26559507 PMC6078167

[B35] Huang C. Zhang Y. Song Y. Wan Z. Wang X. Li R. (2019). Phaeohyphomycosis caused by *Phialophora americana* with CARD9 mutation and 20-year literature review in China. *Mycoses* 62 908–919. 10.1111/myc.12962 31271673

[B36] Jachiet M. Lanternier F. Rybojad M. Bagot M. Ibrahim L. Casanova J.-L. (2015). Posaconazole treatment of extensive skin and nail dermatophytosis due to autosomal recessive deficiency of CARD9. *JAMA Dermatol.* 151 192–194. 10.1001/jamadermatol.2014.2154 25372963

[B37] Jones P. Binns D. Chang H.-Y. Fraser M. Li W. McAnulla C. (2014). InterProScan 5: genome-scale protein function classification. *Bioinformatics* 30 1236–1240. 10.1093/bioinformatics/btu031 24451626 PMC3998142

[B38] Kantarcioglu A. S. Guarro J. de Hoog S. Apaydin H. Kiraz N. (2017). An updated comprehensive systematic review of *Cladophialophora bantiana* and analysis of epidemiology, clinical characteristics, and outcome of cerebral cases. *Med. Mycol.* 55 579–604. 10.1093/mmy/myw124 28007938

[B39] Khaldi N. Seifuddin F. T. Turner G. Haft D. Nierman W. C. Wolfe K. H. (2010). SMURF: genomic mapping of fungal secondary metabolite clusters. *Fungal. Genet. Biol.* 47 736–741. 10.1016/j.fgb.2010.06.003 20554054 PMC2916752

[B40] Kim C. Kim J. Kim S. Cook D. E. Evans K. S. Andersen E. C. (2019). Long-read sequencing reveals intra-species tolerance of substantial structural variations and new subtelomere formation in *C. elegans*. *Genome Res.* 29 1023–1035. 10.1101/gr.246082.118 31123081 PMC6581047

[B41] Koren S. Walenz B. P. Berlin K. Miller J. R. Bergman N. H. Phillippy A. M. (2017). Canu: scalable and accurate long-read assembly via adaptive k-mer weighting and repeat separation. *Genome Res.* 27 722–736. 10.1101/gr.215087.116 28298431 PMC5411767

[B42] Krzywinski M. Schein J. Birol I. Connors J. Gascoyne R. Horsman D. (2009). Circos: an information aesthetic for comparative genomics. *Genome. Res.* 19 1639–1645. 10.1101/gr.092759.109 19541911 PMC2752132

[B43] Lagesen K. Hallin P. Rodland E. A. Staerfeldt H. H. Rognes T. Ussery D. W. (2007). RNAmmer: consistent and rapid annotation of ribosomal RNA genes. *Nucleic Acids Res.* 35 3100–3108. 10.1093/nar/gkm160 17452365 PMC1888812

[B44] Lam K.-K. LaButti K. Khalak A. Tse D. (2015). FinisherSC: a repeat-aware tool for upgrading de novo assembly using long reads. *Bioinformatics* 31 3207–3209. 10.1093/bioinformatics/btv280 26040454

[B45] Lanternier F. Barbati E. Meinzer U. Liu L. Pedergnana V. Migaud M. (2015a). Inherited CARD9 deficiency in 2 unrelated patients with invasive Exophiala infection. *J. Infect. Dis.* 211 1241–1250. 10.1093/infdis/jiu412 25057046 PMC4447834

[B46] Lanternier F. Mahdaviani S. A. Barbati E. Chaussade H. Koumar Y. Levy R. (2015b). Inherited CARD9 deficiency in otherwise healthy children and adults with Candida species-induced meningoencephalitis, colitis, or both. *J. Allergy. Clin. Immunol.* 135 1558–1568. 10.1016/j.jaci.2014.12.1930 25702837 PMC4831587

[B47] Lanternier F. Pathan S. Vincent Q. B. Liu L. Cypowyj S. Prando C. (2013). Deep dermatophytosis and inherited CARD9 deficiency. *N. Engl. J. Med.* 369 1704–1714. 10.1056/NEJMoa1208487 24131138 PMC4084693

[B48] Li H. Durbin R. (2009). Fast and accurate short read alignment with burrows-wheeler transform. *Bioinformatics* 25 1754–1760. 10.1093/bioinformatics/btp324 19451168 PMC2705234

[B49] Li H. Handsaker B. Wysoker A. Fennell T. Ruan J. Homer N. (2009). The sequence alignment/Map format and SAMtools. *Bioinformatics* 25 2078–2079. 10.1093/bioinformatics/btp352 19505943 PMC2723002

[B50] Liao Y. Smyth G. K. Shi W. (2014). FeatureCounts: an efficient general purpose program for assigning sequence reads to genomic features. *Bioinformatics* 30 923–930. 10.1093/bioinformatics/btt656 24227677

[B51] Love M. I. Huber W. Anders S. (2014). Moderated estimation of fold change and dispersion for RNA-seq data with DESeq2. *Genome Biol.* 15 550–550. 10.1186/s13059-014-0550-8 25516281 PMC4302049

[B52] Lowe T. M. Eddy S. R. (1997). tRNAscan-SE: a program for improved detection of transfer RNA genes in genomic sequence. *Nucleic Acids Res.* 25 955–964. 10.1093/nar/25.5.955 9023104 PMC146525

[B53] Lu X. L. Najafzadeh M. J. Dolatabadi S. Ran Y. P. Gerrits van den Ende A. H. G. Shen Y. N. (2013). Taxonomy and epidemiology of *Mucor irregularis*, agent of chronic cutaneous mucormycosis. *Persoonia.* 30 48–56. 10.3767/003158513X665539 24027346 PMC3734966

[B54] Moreno L. F. Ahmed A. A. O. Brankovics B. Cuomo C. A. Menken S. B. J. Taj-Aldeen S. J. (2018). Genomic understanding of an infectious brain disease from the desert. *G3* 8 909–922. 10.1534/g3.117.300421 29326229 PMC5844311

[B55] Price A. L. Jones N. C. Pevzner P. A. (2005). De novo identification of repeat families in large genomes. *Bioinformatics* 21(Suppl. 1) i351–i358. 10.1093/bioinformatics/bti1018 15961478

[B56] Seyedmousavi S. Bosco S. D. M. G. de Hoog S. Ebel F. Elad D. Gomes R. R. (2018). Fungal infections in animals: a patchwork of different situations. *Med. Mycol.* 56 165–187. 10.1093/mmy/myx104 29538732 PMC6251577

[B57] Simão F. A. Waterhouse R. M. Ioannidis P. Kriventseva E. V. Zdobnov E. M. (2015). BUSCO: assessing genome assembly and annotation completeness with single-copy orthologs. *Bioinformatics* 31 3210–3212. 10.1093/bioinformatics/btv351 26059717

[B58] Song Y. Laureijssen-van de Sande W. W. J. Moreno L. F. Gerrits van den Ende B. Li R. de Hoog S. (2017). Comparative ecology of capsular Exophiala species causing disseminated infection in humans. *Front. Microbiol.* 8:2514. 10.3389/fmicb.2017.02514 29312215 PMC5742258

[B59] Vaezi A. Fakhim H. Abtahian Z. Khodavaisy S. Geramishoar M. Alizadeh A. (2018). Frequency and geographic distribution of CARD9 mutations in patients with severe fungal infections. *Front. Microbiol.* 9:2434. 10.3389/fmicb.2018.02434 30369919 PMC6195074

[B60] Walker B. J. Abeel T. Shea T. Priest M. Abouelliel A. Sakthikumar S. (2014). Pilon: an integrated tool for comprehensive microbial variant detection and genome assembly improvement. *PLoS One* 9:e112963. 10.1371/journal.pone.0112963 25409509 PMC4237348

[B61] Wang X. Wang W. Lin Z. Wang X. Li T. Yu J. (2014). *CARD9* mutations linked to subcutaneous phaeohyphomycosis and T_H_17 cell deficiencies. *J. Allergy. Clin. Immunol.* 133 905–908.e3. 10.1016/j.jaci.2013.09.033 24231284

[B62] Yan X. X. Yu C. P. Fu X. A. Bao F. F. Du D. H. Wang C. (2016). CARD9 mutation linked to *Corynespora cassiicola* infection in a Chinese patient. *Br. J. Dermatol.* 174 176–179. 10.1111/bjd.14082 26440558

[B63] Yang Z. (2007). PAML 4: phylogenetic analysis by maximum likelihood. *Mol. Biol. Evol.* 24 1586–1591. 10.1093/molbev/msm088 17483113

[B64] Zhang H. Yohe T. Huang L. Entwistle S. Wu P. Yang Z. (2018). dbCAN2: a meta server for automated carbohydrate-active enzyme annotation. *Nucleic Acids Res.* 46 W95–W101. 10.1093/nar/gky418 29771380 PMC6031026

[B65] Zhang R. Wang X. Wan Z. Li R. (2017). CARD9 mutations and related immunological research of one case with disseminated phaeohyphomycosis. *J. Microbiol. Infect.* 12 14–23.

[B66] Zhao X. Boenisch O. Yeung M. Mfarrej B. Yang S. Turka L. A. (2012). Critical role of proinflammatory cytokine IL-6 in allograft rejection and tolerance. *Am. J. Transplant.* 12 90–101. 10.1111/j.1600-6143.2011.03770.x 21992708

[B67] Zhong X. Chen B. Yang L. Yang Z. (2018). Molecular and physiological roles of the adaptor protein CARD9 in immunity. *Cell. Death Dis.* 9 52–52. 10.1038/s41419-017-0084-6 29352133 PMC5833731

