# Supplementary material for: Comparative Analysis of Clinical and Environmental Strains of Exophiala spinifera by Long-Reads Sequencing and RNAseq Reveal Adaptive Strategies
Source: Front Microbiol. 2020 Jul 31;11:1880. doi: 10.3389/fmicb.2020.01880 (PMC7412599; doi:10.3389/fmicb.2020.01880)
Supplement: Supplementary file 1 [file Table_1.DOCX]

**Table S1.** Overview of genomes of members of Chaetothyriales published to date

| **Species** | **Strain** | **Assembly** | **Size (Mb)** | **GC%** | **Protein** | **rRNA** | **WGS - tRNA** | **WGS - Gene** | **WGS - Pseudogene** | **Assembly name** | **Genome coverage** |
| --- | --- | --- | --- | --- | --- | --- | --- | --- | --- | --- | --- |
| *Arthrocladium fulminans* | CBS 136243 | [QXWJ00000000.1](https://www.ncbi.nlm.nih.gov/nuccore/QXWJ00000000.1) | 27.2 | 51.8 |  |  |  |  |  | Illumina HiSeq | 54x |
| *Cladophialophora bantiana* | CBS 173.52 | [JYBT00000000.1](https://www.ncbi.nlm.nih.gov/nuccore/JYBT00000000.1.) | 36.64 | 51.3 | 12762 | 17 | 38 | 12817 | 4 | Illumina HiSeq | 122x |
| *Cladophialophora carrionii* | CBS 160.54 | [AOFF00000000.1](https://www.ncbi.nlm.nih.gov/nuccore/AOFF00000000.1.) | 28.98 | 54.3 | 10373 | 11 | 44 | 10428 | 8 | Illumina HiSeq | 80x |
| *Cladophialophora immunda* | CBS 834.96 | [JYBZ00000000.1](https://www.ncbi.nlm.nih.gov/nuccore/JYBZ00000000.1.) | 42.97 | 52.8 | 14033 | 15 | 34 | 12879 | 4 | Illumina HiSeq | 143x |
| *Cladophialophora psammophila* | CBS 110553 | [AMGX00000000.1](https://www.ncbi.nlm.nih.gov/nuccore/AMGX00000000.1.) | 39.42 | 50.6 | 13421 | 13421 |  |  |  | Illumina HiSeq | 133x |
| *Cladophialophora yegresii* | CBS 114405 | [AMGW00000000.1](https://www.ncbi.nlm.nih.gov/nuccore/AMGW00000000.1.) | 27.9 | 54 | 10118 | 10118 |  |  |  | Illumina HiSeq | 132x |
| *Exophiala aquamarina* | CBS 119918 | [AMGV00000000.1](https://www.ncbi.nlm.nih.gov/nuccore/AMGV00000000.1.) | 41.57 | 48.3 | 13118 | 13118 |  |  |  | Illumina HiSeq | 79x |
| *Exophiala dermatitidis* | NIH/UT8656 | [AFPA00000000.1](https://www.ncbi.nlm.nih.gov/nuccore/AFPA00000000.1.) | 26.37 | 51.5 | 9578 | 17 | 54 | 9356 | 1 | Illumina HiSeq | 151x |
| *Exophiala mesophila* | CBS 402.95 | [JYBW00000000.1](https://www.ncbi.nlm.nih.gov/nuccore/JYBW00000000.1.) | 29.26 | 50.4 | 10347 | 11 | 45 | 9237 | 5 | Illumina HiSeq | 270x |
| *Exophiala oligosperma* | CBS 725.88 | [JYCA00000000.1](https://www.ncbi.nlm.nih.gov/nuccore/JYCA00000000.1.) | 37.92 | 50.4 | 13234 | 4 | 41 | 11938 | 1 | Illumina HiSeq | 240x |
| *Exophiala sideris* | CBS 121828 | [JYBR00000000.1](https://www.ncbi.nlm.nih.gov/nuccore/JYBR00000000.1.) | 29.48 | 50.7 | 11120 | 15 | 31 | 10237 | 9 | Illumina HiSeq | 271x |
| *Exophiala spinifera* | CBS 899.68 | [JYBY00000000.1](https://www.ncbi.nlm.nih.gov/nuccore/JYBY00000000.1.) | 32.87 | 51.7 | 12049 | 16 | 45 | 12110 | 3 | Illumina HiSeq | 272x |
| *Exophiala xenobiotica* | CBS 118157 | [JYCB00000000.1](https://www.ncbi.nlm.nih.gov/nuccore/JYCB00000000.1.) | 31.39 | 51.5 | 13187 | 13 | 45 | 12077 | 5 | Illumina HiSeq | 87x |
| *Fonsecaea multimorphosa* | CBS 102226 | [JYBV00000000.1](https://www.ncbi.nlm.nih.gov/nuccore/JYBV00000000.1.) | 33.43 | 52.6 | 12369 | 4 | 32 | 12405 | 1 | Illumina HiSeq | 176x |
| *Fonsecaea pedrosoi* | CBS 271.37 | [JYBS00000000.1](https://www.ncbi.nlm.nih.gov/nuccore/JYBS00000000.1.) | 34.63 | 52.4 | 12527 | 11 | 35 | 12573 | 5 | Illumina HiSeq | 115x |
| *Phialophora attae* | CBS 131958 | [LFJN00000000.1](https://www.ncbi.nlm.nih.gov/nuccore/LFJN00000000.1.) | 30.36 | 53.6 | 11848 | 11848 |  |  |  | IonTorrent | 58x |
| *Cyphellophora europaea* | CBS 101466 | [AOBU00000000.1](https://www.ncbi.nlm.nih.gov/nuccore/AOBU00000000.1.) | 28.72 | 54 | 11094 | 14 | 45 | 11153 | 1 | Illumina HiSeq | 132x |
| *Phialophora verrucosa* | BMU07605 | [MSED00000000.1](https://www.ncbi.nlm.nih.gov/nuccore/MSED00000000.1.) | 35.46 | 52.9 |  |  |  |  |  | PacBio | 35x |
| *Rhinocladiella mackenziei* | CBS 650.93 | [JYBU00000000.1](https://www.ncbi.nlm.nih.gov/nuccore/JYBU00000000.1.) | 32.43 | 50.4 | 11382 | 4 | 32 | 11418 | 3 | Illumina HiSeq | 260x |

**Table S3.** Differential expression of single copy genes

| OGID | GeneID | Strains | GeneName | KOG | KOG_description | GOID | GOterm |
| --- | --- | --- | --- | --- | --- | --- | --- |
| OG03091 | ESC1_0211720 | ESE1_0211420 | MFS general substrate transporter | G | Carbohydrate transport and metabolism | GO:0004061\|GO:0016021 | arylformamidase activity\|integral component of membrane\|tryptophan catabolic process to kynurenine\|glyoxylate metabolic process\|transmembrane transport |
| OG07213 | ESC1_0602660 | ESE1_0602580 | uroporphyrinogen decarboxylase | G | Carbohydrate transport and metabolism | GO:0004853\|GO:0005515 | uroporphyrinogen decarboxylase activity\|protein binding\|nucleus\|protoporphyrinogen IX biosynthetic process\|chlorophyll metabolic process\|integral component of membrane\|tRNA methylation\|RNA (guanine-N7)-methylation\|transmembrane transport |
| OG09911 | ESC1_0810310 | ESE1_0117610 | Zip family zinc transporter | P | Inorganic ion transport and metabolism | GO:0016021\|GO:0030001 | integral component of membrane\|metal ion transport\|metal ion transmembrane transporter activity\|transmembrane transport\|NAD+ binding |
| OG07697 | ESC1_0609110 | ESE1_0609430 | predicted protein | Q | Secondary metabolites biosynthesis, transport and catabolism | GO:0005507\|GO:0016491 | copper ion binding\|oxidoreductase activity\|oxidation-reduction process |
| OG01851 | ESC1_0113730 | ESE1_0313310 |  | J | Translation, ribosomal structure and biogenesis |  |  |
| OG02339 | ESC1_0201970 | ESE1_0201920 | O-methyltransferase, putative | S | Function unknown | GO:0008171\|GO:0032259 | O-methyltransferase activity\|methylation |
| OG04825 | ESC1_0401100 | ESE1_0401390 |  |  |  |  |  |
| OG06170 | ESC1_0503400 | ESE1_0503080 | autophagy-like protein 12 | O | Posttranslational modification, protein turnover, chaperones | GO:0000045\|GO:0015031 | autophagosome assembly\|protein transport\|phagophore assembly site membrane |
| OG09004 | ESC1_0712690 | ESE1_0711970 | 60S ribosomal protein L24 | J | Translation, ribosomal structure and biogenesis | GO:0005524\|GO:0005840 | ATP binding\|ribosome\|transferase activity |
| OG04208 | ESC1_0305820 | ESE1_0105780 | uridine kinase | F | Nucleotide transport and metabolism | GO:0004849\|GO:0005524 | uridine kinase activity\|ATP binding\|pyrimidine nucleobase metabolic process\|nucleoside metabolic process\|phosphorylation\|UMP salvage\|CTP salvage |
| OG03224 | ESC1_0213710 | ESE1_0213460 | hypothetical protein PV08_08648 | S | Function unknown | GO:0004601\|GO:0006979 | peroxidase activity\|response to oxidative stress\|integral component of membrane\|cellular oxidant detoxification |
| OG06980 | ESC1_0513890 | ESE1_0514030 | putative endo-1,3(4)-beta-glucanase 2 | E | Amino acid transport and metabolism | GO:0005506\|GO:0016301 | iron ion binding\|kinase activity\|phosphorylation\|oxidoreductase activity, acting on paired donors, with incorporation or reduction of molecular oxygen, reduced flavin or flavoprotein as one donor, and incorporation of one atom of oxygen\|heme binding\|oxidation-reduction process |
| OG02352 | ESC1_0202120 | ESE1_0202080 | WSC domain-containing protein | G | Carbohydrate transport and metabolism | GO:0004553\|GO:0005975 | hydrolase activity, hydrolyzing O-glycosyl compounds\|carbohydrate metabolic process\|integral component of membrane |
| OG07381 | ESC1_0604690 | ESE1_0604690 | putative myosin heavy chain, embryonic smooth muscle isoform | K | Transcription | GO:0005634\|GO:0007030\| | nucleus\|Golgi organization\|negative regulation of transcription by RNA polymerase III |
| OG02554 | ESC1_0205060 | ESE1_0204640 | putative amino-acid N-acetyltransferase subunit Mak10 | S | Function unknown | GO:0017196\|GO:0031417 | N-terminal peptidyl-methionine acetylation\|NatC complex |
| OG01388 | ESC1_0107930 | ESE1_0307490 |  |  |  |  |  |

**Table S8.** A total of 29 genes yielded significant values indicating positive selection

| **OGID** | **ESC1** | **ESE1** | **ESE2** | **lnL0** | **lnL1** | **np0** | **np1** | **model0 dN/dS** | **model2_Clinical**  **dN_dS** | **model2_Environmental**  **dN_dS** | **pvalue** | **FDR** |
| --- | --- | --- | --- | --- | --- | --- | --- | --- | --- | --- | --- | --- |
| OG02735 | ESC1_0207430 | ESE1_0207110 | ESE2_0206990 | -4049.230352 | -4038.585346 | 6 | 7 | 0.70592 | 999.0000 | 0.2605 | 0.0000 | 0.0051 |
| OG03690 | ESC1_0219530 | ESE1_0219640 | ESE2_0219700 | -2856.158209 | -2844.470527 | 6 | 7 | 0.68068 | 999.0000 | 0.1388 | 0.0000 | 0.0025 |
| OG04143 | ESC1_0304870 | ESE1_0104900 | ESE2_0104840 | -5240.018335 | -5232.717766 | 6 | 7 | 0.06194 | 5.8559 | 0.0211 | 0.0001 | 0.0440 |
| OG04461 | ESC1_0308990 | ESE1_0304510 | ESE2_0303940 | -2698.409919 | -2690.386614 | 6 | 7 | 0.43723 | 1.1926 | 0.0001 | 0.0001 | 0.0267 |
| OG05340 | ESC1_0407670 | ESE1_0408060 | ESE2_0408810 | -2252.879048 | -2244.168279 | 6 | 7 | 0.44332 | 0.7845 | 0.0229 | 0.0000 | 0.0170 |
| OG03706 | ESC1_0219730 | ESE1_0219830 | ESE2_0219890 | -6496.221037 | -6483.435054 | 6 | 7 | 0.11032 | 0.6079 | 0.0079 | 0.0000 | 0.0017 |
| OG07670 | ESC1_0608800 | ESE1_0609080 | ESE2_0608870 | -2964.65063 | -2952.984017 | 6 | 7 | 0.39411 | 0.5366 | 0.0001 | 0.0000 | 0.0025 |
| OG08485 | ESC1_0705860 | ESE1_0705260 | ESE2_0705530 | -5511.237005 | -5503.791661 | 6 | 7 | 0.70917 | 0.1980 | 1.3165 | 0.0001 | 0.0440 |
| OG04301 | ESC1_0306900 | ESE1_0107060 | ESE2_0106730 | -4691.150285 | -4681.068322 | 6 | 7 | 0.55894 | 0.0829 | 0.9558 | 0.0000 | 0.0081 |
| OG09125 | ESC1_0714260 | ESE1_0713550 | ESE2_0713770 | -4805.000218 | -4796.974605 | 6 | 7 | 0.63676 | 0.0787 | 0.8688 | 0.0001 | 0.0267 |
| OG09840 | ESC1_0809480 | ESE1_0116740 | ESE2_0116370 | -2519.725552 | -2507.183593 | 6 | 7 | 0.36525 | 0.0161 | 0.6646 | 0.0000 | 0.0017 |
| OG07186 | ESC1_0602370 | ESE1_0202400 | ESE2_0506930 | -5333.104525 | -5325.794652 | 6 | 7 | 0.10911 | 0.0059 | 0.3252 | 0.0001 | 0.0440 |
| OG01016 | ESC1_0102940 | ESE1_0120360 | ESE2_0119850 | -3410.025181 | -3397.237885 | 6 | 7 | 0.22618 | 0.0001 | 0.4427 | 0.0000 | 0.0017 |
| OG01223 | ESC1_0105800 | ESE1_0305260 | ESE2_0304700 | -3501.029167 | -3492.924799 | 6 | 7 | 0.41014 | 0.0001 | 0.6028 | 0.0001 | 0.0267 |
| OG01354 | ESC1_0107560 | ESE1_0307100 | ESE2_0306460 | -3422.436281 | -3412.791083 | 6 | 7 | 0.58613 | 0.0001 | 0.9676 | 0.0000 | 0.0085 |
| OG01391 | ESC1_0107960 | ESE1_0307520 | ESE2_0306880 | -2210.55809 | -2202.389111 | 6 | 7 | 0.08068 | 0.0001 | 999.0000 | 0.0001 | 0.0267 |
| OG01496 | ESC1_0109190 | ESE1_0308780 | ESE2_0308170 | -2864.211421 | -2854.550696 | 6 | 7 | 0.2475 | 0.0001 | 0.3703 | 0.0000 | 0.0085 |
| OG01751 | ESC1_0112340 | ESE1_0311960 | ESE2_0311380 | -1968.804232 | -1961.557756 | 6 | 7 | 0.16542 | 0.0001 | 0.6570 | 0.0001 | 0.0440 |
| OG02765 | ESC1_0207790 | ESE1_0207470 | ESE2_0207330 | -3135.228629 | -3126.260923 | 6 | 7 | 0.63684 | 0.0001 | 0.8565 | 0.0000 | 0.0148 |
| OG04065 | ESC1_0303990 | ESE1_0103970 | ESE2_0103960 | -6657.493309 | -6647.765239 | 6 | 7 | 0.35337 | 0.0001 | 0.6825 | 0.0000 | 0.0085 |
| OG05036 | ESC1_0403660 | ESE1_0404030 | ESE2_0404690 | -2358.180423 | -2348.976268 | 6 | 7 | 0.56535 | 0.0001 | 0.8283 | 0.0000 | 0.0125 |
| OG06252 | ESC1_0504320 | ESE1_0504020 | ESE2_0504100 | -5784.279299 | -5773.14447 | 6 | 7 | 0.61632 | 0.0001 | 0.7225 | 0.0000 | 0.0036 |
| OG06300 | ESC1_0505060 | ESE1_0504830 | ESE2_0504900 | -2241.376319 | -2233.788244 | 6 | 7 | 0.37453 | 0.0001 | 0.4971 | 0.0001 | 0.0404 |
| OG07937 | ESC1_0612400 | ESE1_0612370 | ESE2_0612260 | -1178.311615 | -1170.956508 | 6 | 7 | 0.17534 | 0.0001 | 1.0821 | 0.0001 | 0.0440 |
| OG08169 | ESC1_0701490 | ESE1_0701490 | ESE2_0701810 | -4815.341032 | -4806.931684 | 6 | 7 | 0.42903 | 0.0001 | 0.9352 | 0.0000 | 0.0220 |
| OG09014 | ESC1_0712830 | ESE1_0712100 | ESE2_0712360 | -2429.608061 | -2422.203463 | 6 | 7 | 0.39028 | 0.0001 | 2.5866 | 0.0001 | 0.0440 |
| OG09312 | ESC1_0802550 | ESE1_0110340 | ESE2_0110000 | -3669.180918 | -3660.367413 | 6 | 7 | 0.39397 | 0.0001 | 0.6566 | 0.0000 | 0.0163 |
| OG09661 | ESC1_0807030 | ESE1_0114540 | ESE2_0114140 | -3901.964347 | -3892.25753 | 6 | 7 | 0.3584 | 0.0001 | 0.9010 | 0.0000 | 0.0085 |
| OG09841 | ESC1_0809490 | ESE1_0116750 | ESE2_0116390 | -4989.972162 | -4982.708089 | 6 | 7 | 0.37186 | 0.0001 | 0.4881 | 0.0001 | 0.0440 |

**Table S9.** GO enrichment on positive selection genes

| **GO ID** | **Term** | **Annotated genes** | **Significant genes** | **Expected** | **P-value** | **Ontology** |
| --- | --- | --- | --- | --- | --- | --- |
| GO:0016558 | protein import into peroxisome matrix | 5 | 1 | 0.02 | 0.015 | BP |
| GO:0034724 | DNA replication-independent nucleosome organization | 5 | 1 | 0.02 | 0.015 | BP |
| GO:0042327 | positive regulation of phosphorylation | 5 | 1 | 0.02 | 0.015 | BP |
| GO:0006970 | response to osmotic stress | 5 | 1 | 0.02 | 0.015 | BP |
| GO:0000165 | MAPK cascade | 6 | 1 | 0.02 | 0.018 | BP |
| GO:0071214 | cellular response to abiotic stimulus | 6 | 1 | 0.02 | 0.018 | BP |
| GO:0051347 | positive regulation of transferase activity | 6 | 1 | 0.02 | 0.018 | BP |
| GO:0009408 | response to heat | 7 | 1 | 0.02 | 0.021 | BP |
| GO:0006835 | dicarboxylic acid transport | 7 | 1 | 0.02 | 0.021 | BP |
| GO:1902533 | positive regulation of intracellular signal transduction | 8 | 1 | 0.02 | 0.024 | BP |
| GO:0005524 | ATP binding | 633 | 6 | 2.02 | 0.012 | MF |
| GO:0004674 | protein serine/threonine kinase activity | 66 | 2 | 0.21 | 0.018 | MF |
| GO:0005310 | dicarboxylic acid transmembrane transporter activity | 6 | 1 | 0.02 | 0.019 | MF |
| GO:0031491 | nucleosome binding | 7 | 1 | 0.02 | 0.022 | MF |
| GO:0042393 | histone binding | 9 | 1 | 0.03 | 0.028 | MF |
| GO:0016878 | acid-thiol ligase activity | 11 | 1 | 0.04 | 0.035 | MF |
| GO:0061630 | ubiquitin protein ligase activity | 13 | 1 | 0.04 | 0.041 | MF |
| GO:0071014 | post-mRNA release spliceosomal complex | 5 | 1 | 0.02 | 0.02 | CC |
| GO:0043596 | nuclear replication fork | 6 | 1 | 0.02 | 0.024 | CC |
| GO:0030127 | COPII vesicle coat | 6 | 1 | 0.02 | 0.024 | CC |
| GO:0045239 | tricarboxylic acid cycle enzyme complex | 8 | 1 | 0.03 | 0.032 | CC |
| GO:0030119 | AP-type membrane coat adaptor complex | 9 | 1 | 0.04 | 0.036 | CC |
| GO:0005684 | U2-type spliceosomal complex | 10 | 1 | 0.04 | 0.04 | CC |
| GO:0008023 | transcription elongation factor complex | 10 | 1 | 0.04 | 0.04 | CC |
| GO:0005730 | nucleolus | 88 | 2 | 0.36 | 0.05 | CC |
