# Supplementary figures and images for: Comparative Analysis of Clinical and Environmental Strains of Exophiala spinifera by Long-Reads Sequencing and RNAseq Reveal Adaptive Strategies
Source: Front Microbiol. 2020 Jul 31;11:1880. doi: 10.3389/fmicb.2020.01880 (PMC7412599; doi:10.3389/fmicb.2020.01880)

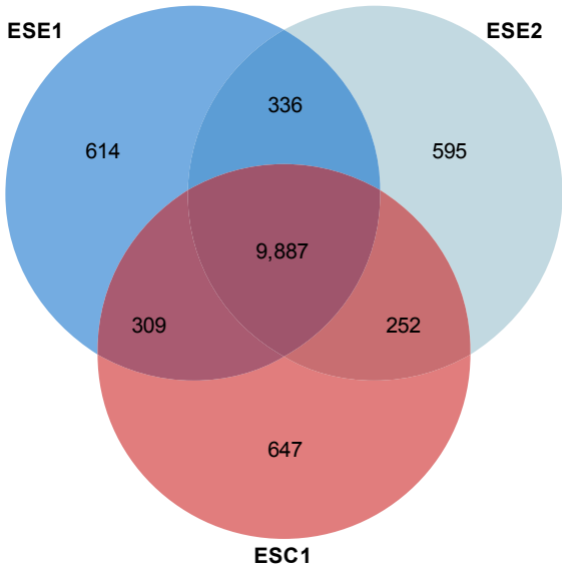

Supplement: Supplementary file 7 [file Image_1.PDF]

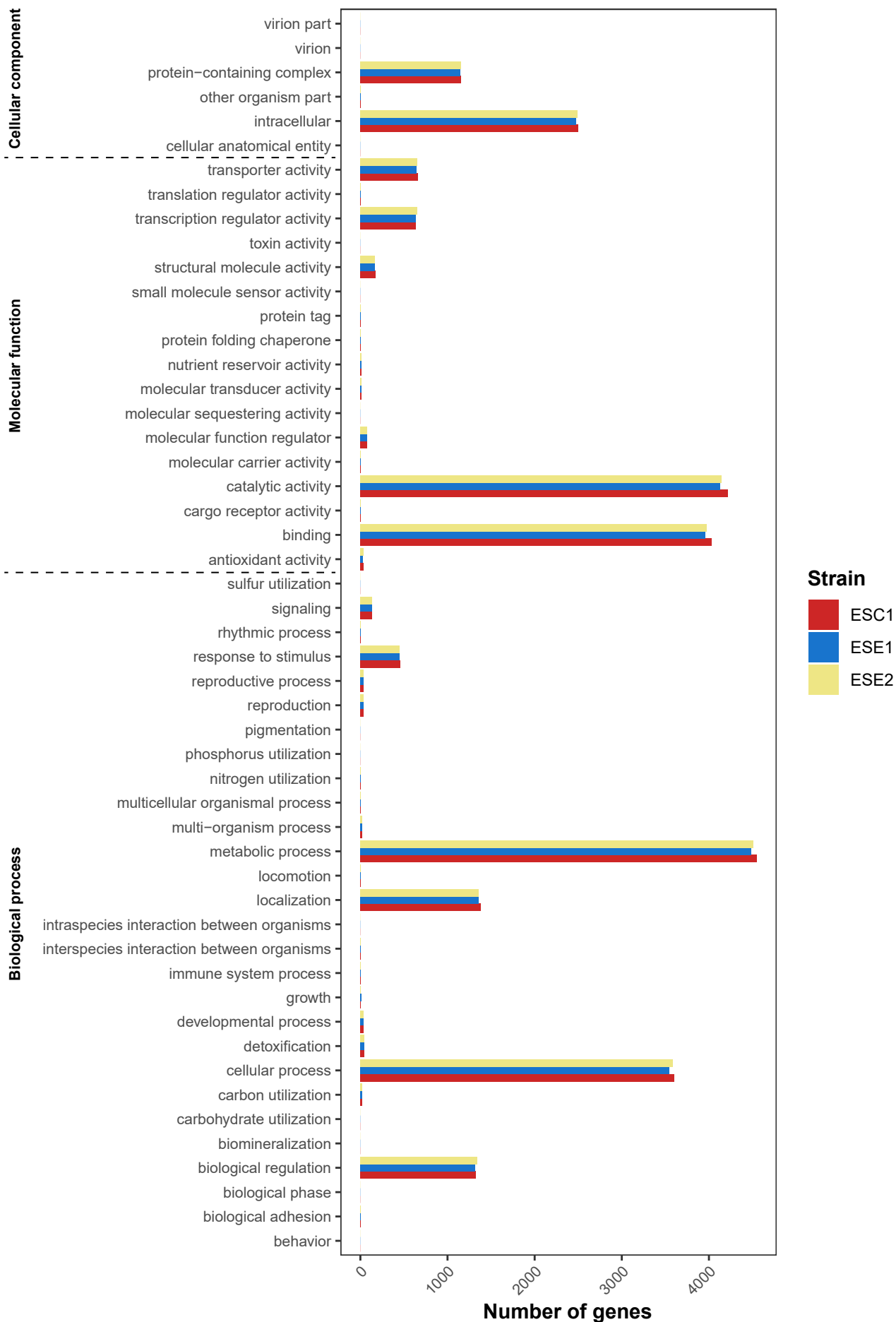

Supplement: Supplementary file 8 [file Image_2.PDF]

Term

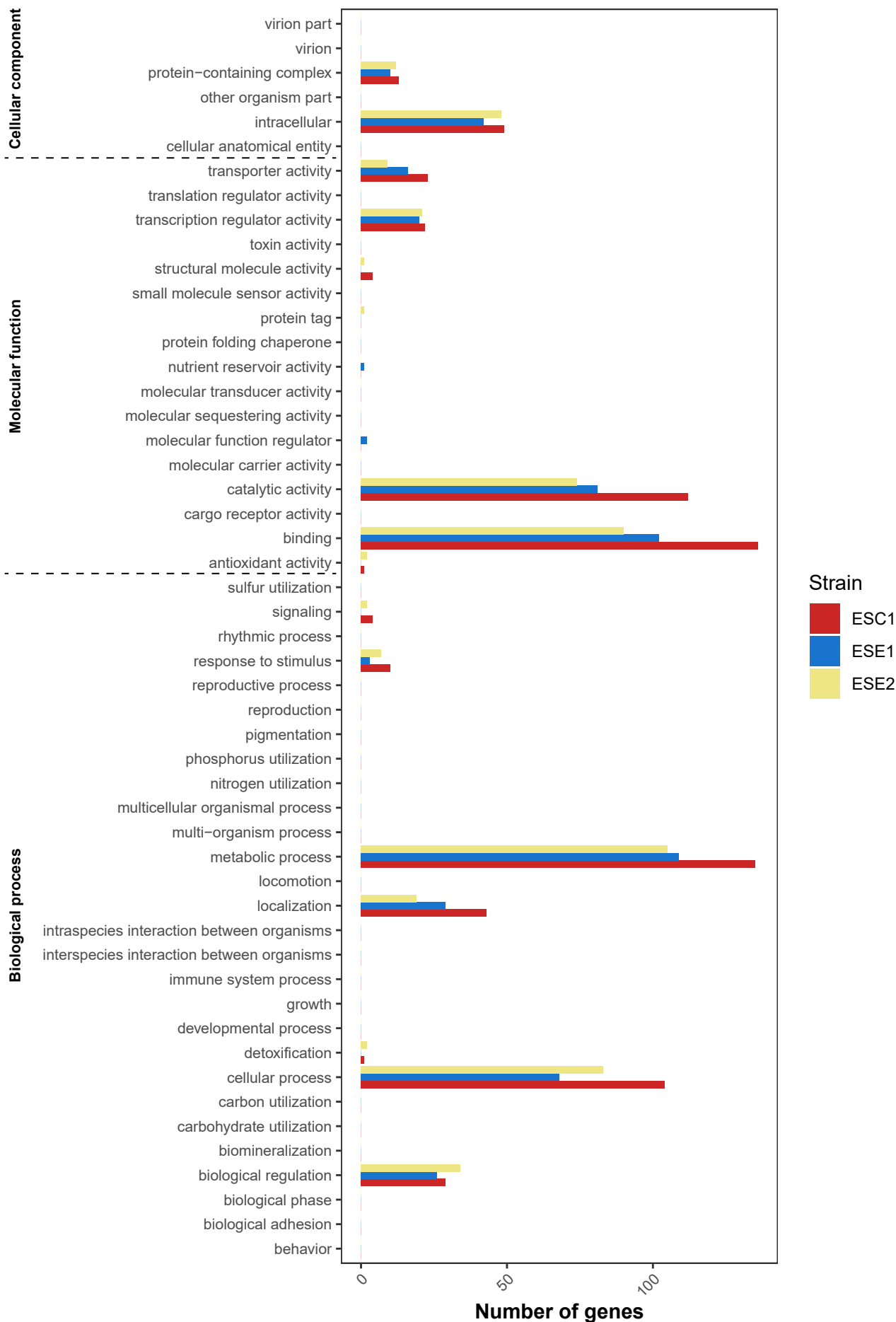

Supplement: Supplementary file 9 [file Image_3.PDF]
